# Standard-Dose Proton Pump Inhibitors in the Initial Non-eradication Treatment of Duodenal Ulcer: Systematic Review, Network Meta-Analysis, and Cost-Effectiveness Analysis

**DOI:** 10.3389/fphar.2018.01512

**Published:** 2019-01-07

**Authors:** Jiaxing Zhang, Long Ge, Matt Hill, Yi Liang, Juan Xie, Dejun Cui, Xiaosi Li, Wenyi Zheng, Rui He

**Affiliations:** ^1^Department of Pharmacy, Guizhou Provincial People’s Hospital, Guiyang, China; ^2^First Clinical Medical College, Lanzhou University, Lanzhou, China; ^3^Health Outcomes and Pharmacy Practice, College of Pharmacy, The University of Texas at Austin, Austin, TX, United States; ^4^Department of Gastroenterology, Guizhou Provincial People’s Hospital, Guiyang, China; ^5^Department of Pharmacy, Hospital of Chengdu Office of People’s Government of Tibetan Autonomous Region, Chengdu, China; ^6^Experimental Cancer Medicine, Clinical Research Center, Department of Laboratory Medicine, Karolinska Institute, Stockholm, Sweden

**Keywords:** proton pump inhibitors, duodenal ulcer, systematic review, network meta-analysis, cost-effectiveness analysis

## Abstract

**Background:** Short-term use of standard-dose proton pump inhibitors (PPIs) is the first-line initial non-eradication treatment for duodenal ulcer (DU), but the choice on individual PPI drug is still controversial. The purpose of this study is to compare the efficacy, safety, and cost-effectiveness of standard-dose PPI medications in the initial non-eradication treatment of DU.

**Methods:** We searched PubMed, Embase, Cochrane Library, Clinicaltrials.gov, China National Knowledge Infrastructure, VIP database, and the Wanfang database from their earliest records to September 2017. Randomized controlled trials (RCTs) evaluating omeprazole (20 mg/day), pantoprazole (40 mg/day), lansoprazole (30 mg/day), rabeprazole (20 mg/day), ilaprazole (10 mg/day), ranitidine (300 mg/day), famotidine (40 mg/day), or placebo for DU were included. The outcomes were 4-week ulcer healing rate (4-UHR) and the incidence of adverse events (AEs). A network meta-analysis (NMA) using a Bayesian random effects model was conducted, and a cost-effectiveness analysis using a decision tree was performed from the payer’s perspective over 1 year.

**Results:** A total of 62 RCTs involving 10,339 participants (eight interventions) were included. The NMA showed that all the PPIs significantly increased the 4-UHR compared to H_2_ receptor antagonists (H_2_RA) and placebo, while there was no significant difference for 4-UHR among PPIs. As to the incidence of AEs, no significant difference was observed among PPIs, H_2_RA, and placebo during 4-week follow-up. Based on the costs of both PPIs and management of AEs in China, the incremental cost-effectiveness ratio per quality-adjusted life year (in US dollars) for pantoprazole, lansoprazole, rabeprazole, and ilaprazole compared to omeprazole corresponded to $5134.67, $17801.67, $25488.31, and $44572.22, respectively.

**Conclusion:** Although the efficacy and tolerance of different PPIs are similar in the initial non-eradication treatment of DU, pantoprazole (40 mg/day) seems to be the most cost-effective option in China.

## Introduction

Duodenal ulcer (DU) is a defect in the duodenal wall that extends through the muscularis mucosa into the deeper layers (Editorial Board of Chinese Journal of Digestion, 2016). A systematic review (Lin et al., 2011) estimated that the pooled incidence rate of uncomplicated DU, DU bleeding, and perforated DU was 0.510 (95% confidence interval: 0.380–0.670), 0.240(0.190–0.300), and 0.055 (0.038–0.079) per 1,000 person-years, respectively. The prevalence of DU reported in recent population-based studies varied greatly in different countries: 2.1% in Sweden (Aro et al., 2006), 3.9% in Italy (Zagari et al., 2010), 5.6% in Northern Saudi Arabia (Albaqawi et al., 2017), 7.4% in Bangladesh (Ghosh et al., 2017), and 13.3% in China (Li et al., 2010), respectively.

Although eradication of *Helicobacter pylori* (Hp) is associated with higher healing rates and lower ulcer recurrence rates in patients with Hp-positive DU (Leodolter et al., 2001; Ford et al., 2016), non-eradication therapies are still appropriate for the patients with Hp-negative DU or without the result of Hp testing. Pump proton inhibitors (PPIs) are a kind of benzimidazole prodrug that inhibit gastric acid secretion by irreversibly binding to the hydrogen-potassium ATPase pump residing on the luminal surface of the parietal cell membrane (Wolfe and Sachs, 2000; Shin et al., 2004). These agents have been recommended by the Japanese Society of Gastroenterology (JSG) as first-line treatment for the initial non-eradication treatment of DU (Satoh et al., 2016). Chinese guidelines recommended the standard dose of PPI given over 4–6 weeks for the treatment of DU (Editorial Board of Chinese Journal of Digestion, 2016). Omeprazole (OME; 20 mg/day), lansoprazole (LAN; 30 mg/day), pantoprazole (PAN; 40 mg/day), rabeprazole (RAB; 20 mg/day), ilaprazole (ILA; 10 mg/day), and esomeprazole (ESO; 20 mg/day) are widely used PPIs in the initial non-eradication treatment of DU. PPIs differ in their pKa, bioavailability, peak plasma levels, and route of excretion. A previous network meta-analysis (Hu et al., 2017) of randomized controlled trials (RCTs) compared the healing rates and adverse effects of different PPIs in ordinary doses for patients with DU and concluded there was no significant difference for the efficacy and tolerance between the ordinary doses of PPIs. However, this study included 24 RCTs and compared nine interventions, which resulted in an underpowered test. Moreover, ranitidine (RAN) and famotidine (FAM) were considered one intervention (H_2_RA), which introduced clinical heterogeneity to the model. Therefore, this conclusion needs to be further verified. On the other hand, cost-effectiveness among PPIs is still controversial due to high variability in cost. The present study aims to evaluate the efficacy, safety, and cost-effectiveness of standard-dose PPI medications in the initial non-eradication treatment of DU.

## Materials and Methods

We followed the PRISMA Extension Statement for Reporting of Systematic Reviews Incorporating Network Meta-analyses of Health Care Interventions (Supplementary Table S1). The systematic review was prospectively registered on International Prospective Register of Systematic Review (PROSPERO, CRD42017079704). The economic evaluation reporting also followed the Consolidated Health Economic Evaluation Reporting Standards Statement (CHEERS) (Supplementary Table S2).

### Search

PubMed, Embase, and the Cochrane Central Register of Controlled Trials (CENTRAL) were searched using the search strategies detailed in Supplementary Table S3, from their inception to September 2017. Clinicaltrials.gov also was searched using the terms “duodenal ulcer,” “proton pump inhibitor,” “omeprazole,” “pantoprazole,” “lansoprazole,” “rabeprazole,” “ilaprazole,” “esomeprazole,” “famotidine,” and “ranitidine.” The China National Knowledge Infrastructure (CNKI), VIP database, and Wanfang database were also searched with Chinese terms. We reviewed the references from published network meta-analyses of PPIs, included studies, and relevant review articles to find additional studies.

### Eligibility Criteria

We included studies meeting the following criteria: (1) RCTs; (2) participants with endoscopically verified DU; (3) a focus on the following interventions by oral administration: OME 20 mg/day, PAN 40 mg/day, LAN 30 mg/day, RAB 20 mg/day, ILA 10 mg/day, ESO 20 mg/day, RAN 300 mg/day, FAM 40 mg/day, and placebo (PLA); (4) the duration of treatment should be 4 weeks or longer; (5) Reporting on any of the following outcomes: 4-week ulcer healing rate (4-UHR, primary outcome), defined as complete re-epithelialization of the ulcer crater irrespective of residual erosions after 4 weeks of treatment; incidence of overall adverse events (AEs, secondary outcome); and (6) published in English or Chinese.

We excluded studies that enrolled participants with upper gastrointestinal bleeding, stress ulcer, or the concomitant therapy for Hp eradication, studies compared only different doses of the same drug, and studies reported as in-conference abstracts, which were impossible to assess the risk of bias.

### Study Selection and Data Extraction

Two reviewers independently screened the titles and abstracts of all studies identified by the search strategies according to the inclusion criteria. The full-texts of all potentially relevant articles were downloaded for further reviewing. We resolved any disagreements through discussion or adjudication by a third reviewer (Juan Xie).

We used a pre-designed data collection form to extract data from each eligible study, including: (1) authors, year of publication, country or region where the study conducted; (2) study design; (3) medication used in treatment or control group, dose, and duration of treatment; (4) number of participants randomized into each group; (5) diagnosis, gender, age, smoking and drinking habits of participants; (6) length of follow up; (7) outcome data (outcomes of interest, events and number of patients included for analyses in each group); and (8) sources of funding. As to the outcome data, we extracted intention-to-treat (ITT) data where these were reported. Otherwise, we extracted the data as reported (often a modified ITT based on, e.g., all patients who received at least one dose of the study drug). A kappa statistic (K) was manually calculated to measure the agreement between two reviewers on the decisions made in study selection.

### Risk of Bias Assessment

Two reviewers independently assessed the risk of bias in each included study using the tool developed by Cochrane Collaboration (Higgins and Green, 2011). The items included random sequence generation, allocation concealment, blinding, incomplete outcome data, selective outcome reporting, and other bias. We categorized the judgments as low, high, or unclear risk of bias and created a summary graph using Review Manager Software (version 5.3).

### Statistical Synthesis

We generated network plots of comparisons to illustrate which interventions had been compared within randomized trials (head-to-head comparisons). A Bayesian random effects network meta-analysis was conducted to compare the relative efficacy (4-UHR) and safety (the incidence of AEs) between different regimens. WinBUGS (version 1.4.3) was used to perform the analysis. Posterior samples were generated using Markov Chain Monte-Carlo (MCMC) simulation in two parallel chains. We used 5,000 burn-in iterations to allow convergence, and then a further 50,000 iterations to produce the outputs. We calculated odds ratios (ORs) with 95% confidence intervals (95% CIs), and a surface under the cumulative ranking (SUCRA). We evaluated and graded the statistical heterogeneity according to the value of *I*^2^. A value for *I*^2^ of 50% or greater was used to denote significant heterogeneity. A node-splitting approach employed to assess inconsistency in the triangular loop (van Valkenhoef et al., 2016) using the gemtc package in the R environment (version 3.3.1) (van Valkenhoef et al., 2012). In order to observe the robustness of results, we conducted sensitivity analysis to compare the results from ITT data to per-protocol (PP) data. We also conducted a sensitive analysis by excluding trials with high risk of bias. Subgroup analyses were also conducted between Chinese and non-Chinese participants. Patients from Chinese Mainland, Hong Kong and Taiwan were considered to be Chinese for this study.

### Cost-Effectiveness Analysis

We evaluated the cost-effectiveness of PPIs in Chinese patients with DU from the payer’s perspective. A decision tree model was constructed in Excel to explore the economic benefits and Quality-Adjusted Life Year (QALY) gains. The model considered costs and outcomes over 1 year, and was based on 10000 Chinese DU patients (male/female = 1), one each in the OME, PAN, LAN, RAB, ILA, and ESO arms. To estimate the probability of 4-UHR for OME, we conducted a single arm meta-analysis based on data from trials on OME with a random-effect model using the meta package in the R environment (version 3.3.1) (DerSimonian and Laird, 1986). Then the probability for OME and the OR for 4-UHR for each PPI versus OME as estimated in the NMA were employed to produce the respective probabilities for other PPIs. To estimate QALYs, we extracted the data about health state utility value from previously published research (Groeneveld et al., 2001; Sun et al., 2011). The cost of each treatment strategy was calculated according to the drug cost for one standard treatment (4 weeks) obtained from the National Health and Family Planning Commission of the People’s Republic of China^1^. The costs of managing AEs were obtained from the published literature (Xuan et al., 2016), while all other costs associated with administering the medications were assumed to be the same across the five arms. All costs were recorded in Chinese yuan and then converted into US dollars (exchange rate: 1 yuan = $0.1591). The incremental cost-effectiveness ratio (ICER) per additional life-years saved was calculated to compare the performance of different PPIs. We considered treatment strategies with an ICER of less than $25,761 (i.e., 3-times Chinese gross domestic product [GDP] (Hutubessy et al., 2003) per capita in 2016^2^) per QALY saved to be acceptable. Probabilistic sensitivity analysis (PSA) was performed to test the robustness of the model.

## Results

### Search Result and Characteristics of Included Studies

**FIGURE 1:**
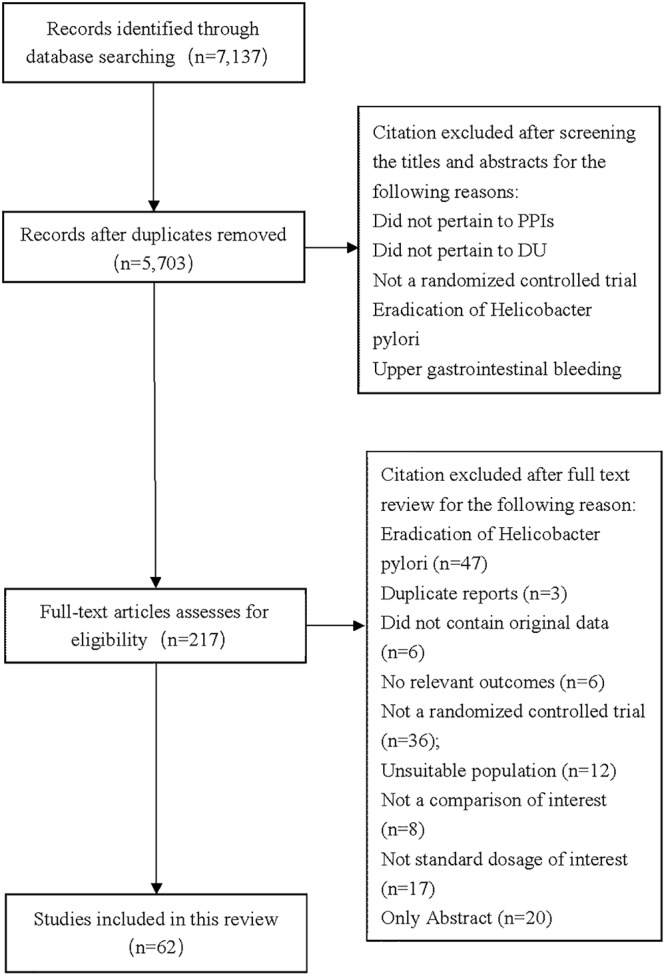
Flow diagram of study selection process for this systematic review.

**FIGURE 2:**
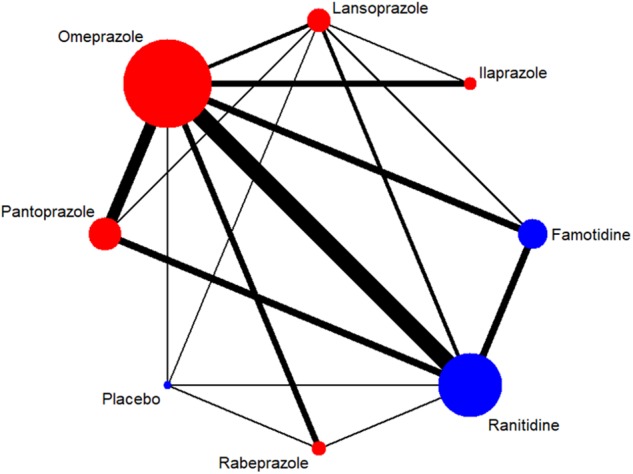
Network of included RCTs with available direct comparisons for 4-week ulcer healing rate. The widths of the lines indicate the number of included trials.

**FIGURE 3:**
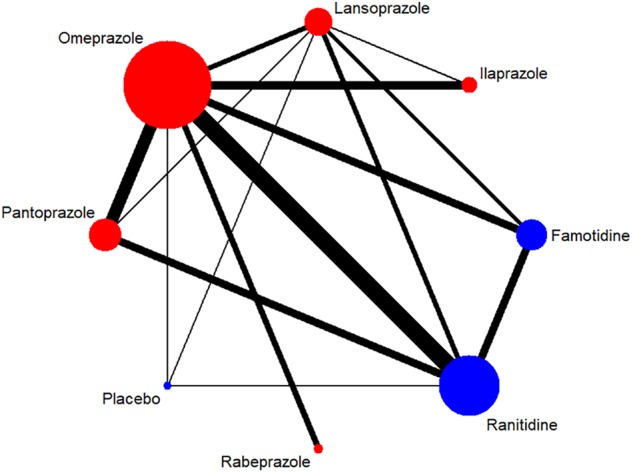
Network of included RCTs with available direct comparisons for adverse events. The widths of the lines indicate the number of included trials.

A total of 7,137 citations were obtained from the literature search (*K* = 0.9). Figure 1 showed the selection process. Sixty-two RCTs (Barbara et al., 1985; Simon et al., 1985; Bardhan et al., 1986; Mccullough, 1986; Dobrilla et al., 1987; Marks and Wright, 1987; Alcalá-Santaella et al., 1989; Chelvam et al., 1989; Hui et al., 1989; Mulder et al., 1989; Graham et al., 1990; McFarland et al., 1990; Londong et al., 1991; Marks et al., 1991; Valenzuela et al., 1991; Delle Fave et al., 1992; Hotz et al., 1992; Kumar et al., 1992; Lysy et al., 1992; Wang et al., 1992, 2000, 2011, 2012; Ahmed et al., 1993; Tao and Yin, 1993; Hawkey et al., 1993; Misra et al., 1993; Zaterka et al., 1993; Judmaier and Koelz, 1994; Lanza et al., 1994; Li et al., 1994, 2001, 2010; Rensburg et al., 1994; Chang et al., 1995; Cremer et al., 1995; Ekström et al., 1995; Rehner et al., 1995; Schepp and Classen, 1995; Xiao, 1997; Cloud et al., 1998; Hallerback et al., 1998; Dekkers et al., 1999; Breiter et al., 2000; Meneghelli et al., 2000; Pei et al., 2000; Clinical Study Group of Pantoprazole in Shanghai, 2001; Hu, 2001; Tang and Hu, 2001; Xu, 2001, 2006; Gu, 2005; You et al., 2006; Ho et al., 2009; Huang, 2010; Liu and Cheng, 2011; Zou, 2012; Wang and Chen, 2013; Zhao et al., 2013; Liao, 2015; Chen, 2017; Chen et al., 2017) with 10,339 participants were included in the network meta-analysis (Li and Li, 2010). As shown in Supplementary Table S4, included trials were conducted in 27 countries or regions (e.g., Australia, Belgium, Brazil, Canada, China, France, Germany, Hong Kong, Iceland, India, Ireland, Israel, Italy, Karachi, Malaysia, Netherlands, Philippines, Poland, Singapore, South Africa, Spain, Sweden, Switzerland, Taiwan, Thailand, the United Kingdom, and the United States). There were 39 trials published in English and 23 trials published in Chinese, respectively. Among the included trials, 60 were two-arm studies and 2 were three-arm studies, with a total of eight different interventions (Figures 2, 3). No study regarding ESO was included according to the inclusion and exclusion criteria (e.g., studies reported as in-conference abstracts, studies with the concomitant therapy for Hp eradication, or studies for gastric ulcer). The baseline characteristics of the participants were shown in Supplementary Table S4. The age of participants was ranged from 16 to 85 years old, and the proportion of males was ranged from 43.33 to 92.79%. Thirty-eight studies reported the number of smokers (28.13–72.46%). Twenty-eight studies reported the number of participants drinking alcohol (0.90–68.19%). The duration of treatment ranged from 4 to 8 weeks.

### Risk of Bias Assessment

As shown in Supplementary Figure S1, four studies (Delle Fave et al., 1992; Rensburg et al., 1994; Meneghelli et al., 2000; Hu, 2001) had low risk of selection bias for clearly describing the methods of randomization and allocation concealment, while the other 58 were unclear because the information about selection participants was not reported. Thirty-nine studies (62.90%) had low risk of performance bias and detection bias, as both participants and study personnel were masked; however, this risk was not clear in 23 studies (37.10%) for failing to report who was blinded. Sixty-one studies (98.39%) had low risk of attrition bias, as there was no loss to follow-up or missing data was appropriately addressed (e.g., applying ITT analysis which could underestimate the efficacy of the intervention). Thirty-nine studies (62.90%) had low risk of reporting bias since they had reported all predesigned outcomes. The other 23 studies (37.10%) neither mentioned registration information nor had an available protocol, so it was unclear whether all the pre-designed outcomes in these studies had been reported. Eight studies (12.90%) were supported by pharmaceutical industry, and bias caused by conflict of interest was unclear.

### Four-Week Ulcer Healing Rate (4-UHR)

The 4-UHR was reported in 62 studies including 10,339 patients. There was no substantial heterogeneity among studies across most comparisons (*I*^2^< 50%), with the exception of RAN vs. PAN (*I*^2^= 59.5%) (Supplementary Figure S2). Inconsistency was not detected across all comparison groups (*P* > 0.05) (Supplementary Figure S3). Table 1 showed that all the PPIs significantly improved healing rates compared to H_2_RAs and PLA, while there was no significant difference among PPIs. The probability of each regimen to be the most superior was shown in Supplementary Table S5, which indicated that ILA (SUCRA = 1.0, 95% CI: 0.7143–1.0), PAN (SUCRA = 0.8571, 95% CI: 0.5714–1.0), RAB (SUCRA = 0.5714, 95% CI: 0.4286–1.0), OME (SUCRA = 0.5714, 95% CI: 0.4286–0.8571), and LAN (SUCRA = 0.4286, 95% CI: 0.4286–0.8571) were the top five regimens, respectively.

**Table 1:** Odds ratio of various PPIs from the network meta-analysis for 4-week ulcer healing rate (intention-to-treat data).

OR	OME	PAN	LAN	RAB	ILA	RAN	FAM	PLA
OME	1.0	11.241 (0.942, 1.655)	10.900 (0.634, 1.268)	10.988 (0.596, 1.638)	11.463 (0.994, 2.185)	10.420 (0.348, 0.511)	10.328 (0.254, 0.424)	10.152 (0.093, 0.258)
PAN	10.806 (0.604, 1.064)	1.0	10.721 (0.477, 1.091)	10.802 (0.457, 1.366)	11.181 (0.732, 1.922)	10.339 (0.259, 0.441)	10.265 (0.189, 0.371)	10.121 (0.072, 0.219)
LAN	11.110 (0.789, 1.576)	11.386 (0.917, 2.099)	1.0	11.110 (0.615, 1.960)	11.648 (0.989, 2.702)	10.471 (0.331, 0.650)	10.366 (0.251, 0.536)	10.170 (0.099, 0.303)
RAB	11.013 (0.611, 1.677)	11.247 (0.732, 2.188)	10.901 (0.510, 1.626)	1.0	11.457 (0.801, 2.859)	10.421 (0.261, 0.694)	10.334 (0.192, 0.563)	10.153 (0.083, 0.282)
ILA	10.684 (0.458, 1.006)	10.847 (0.520, 1.366)	10.607 (0.370, 1.012)	10.686 (0.350, 1.248)	1.0	10.288 (0.185, 0.445)	10.225 (0.138, 0.358)	10.103 (0.055, 0.199)
RAN	12.381 (1.958, 2.876)	12.949 (2.265, 3.860)	12.122 (1.539, 3.024)	12.375 (1.440, 3.841)	13.472 (2.248, 5.416)	1.0	10.780 (0.621, 0.994)	10.359 (0.221, 0.614)
FAM	13.052 (2.359, 3.940)	13.773 (2.694, 5.302)	12.729 (1.867, 3.979)	12.993 (1.777, 5.201)	14.440 (2.794, 7.243)	11.281 (1.006, 1.611)	1.0	10.455 (0.270, 0.815)
PLA	16.599 (3.877, 10.770)	18.261 (4.565, 13.940)	15.891 (3.306, 10.150)	16.518 (3.549, 12.050)	19.709 (5.028, 18.250)	12.787 (1.630, 4.519)	12.197 (1.227, 3.701)	1.0


*Each number is an odds ratio (=row/column), and 95% confidence interval. OME, omeprazole, 20 mg/day; PAN, pantoprazole, 40 mg/day; LAN, lansoprazole, 30 mg/day; RAB, rabeprazole, 20 mg/day; ILA, ilaprazole, 10 mg/day; RAN, ranitidine, 300 mg/day; FAM, famotidine, 40 mg/day; PLA, placebo. Green shading: no significant difference; red shading: significant difference.*

### Incidence of AEs

Fifty studies (9,012 participants) reported the overall incidence of any AEs in participants receiving the eight interventions. The heterogeneity (Supplementary Figure S4) was not statistically significant among most comparisons (*I*^2^< 50%), except for PAN vs. LAN (*I*^2^= 51.8% for network). The inconsistency (Supplementary Figure S5) was also not statistically significant among most triangular loops with exception of PAN vs. OME (*P* = 0.0359). As shown in Table 2, there was no significant difference for the incidence of AEs among all the PPIs, H_2_RAs, and PLA. The results of SUCRA (Supplementary Table S5) indicated that the relative ranking of preferred agents for safety was: ILA (0.8571, 95% CI: 0.2857–1.0), RAB (0.8571, 95% CI: 0.0–1.0), LAN (0.5714, 95% CI: 0.1429–1.0), FAM (0.5714, 95% CI: 0.0–1.0), OME (0.4286, 95% CI: 0.1429–0.8571), RAN (0.4286, 95% CI: 0.1429–0.8571), PAN (0.2857, 95% CI: 0.0–0.8571), PLA (0.0, 95% CI: 0.0–0.8571). The most common AEs included headache, diarrhea, nausea, rash, vomiting, dizziness, constipation, and myalgia (Table 3). Most AEs were mild, transient, and reversible.

**Table 2:** Odds ratio of various PPIs from the network meta-analysis for adverse events.

OR	OME	PAN	LAN	RAB	ILA	RAN	FAM	PLA
OME	1.0	11.074 (0.720, 1.602)	10.905 (0.618, 1.366)	10.778 (0.390, 1.465)	10.711 (0.440, 1.105)	10.983 (0.748, 1.281)	10.895 (0.565, 1.432)	11.659 (0.714, 3.676)
PAN	10.931 (0.624, 1.390)	1.0	10.844 (0.511, 1.437)	10.732 (0.328, 1.543)	10.661 (0.363, 1.186)	10.915 (0.621, 1.334)	10.834 (0.474, 1.480)	11.548 (0.6176, 3.669)
LAN	11.105 (0.732, 1.619)	11.185 (0.696, 1.957)	1.0	10.865 (0.381, 1.770)	10.780 (0.430, 1.386)	11.079 (0.701, 1.612)	10.982 (0.598, 1.614)	11.821 (0.724, 4.295)
RAB	11.285 (0.683, 2.564)	11.366 (0.648, 3.045)	11.157 (0.565, 2.622)	1.0	10.915 (0.417, 2.078)	11.261 (0.636, 2.658)	11.142 (0.528, 2.660)	12.130 (0.746, 6.160)
ILA	11.406 (0.905, 2.276)	11.512 (0.844, 2.753)	11.282 (0.722, 2.327)	11.093 (0.481, 2.397)	1.0	11.389 (0.819, 2.355)	11.261 (0.665, 2.420)	12.350 (0.911, 5.847)
RAN	11.017 (0.781, 1.338)	11.093 (0.750, 1.610)	10.927 (0.620, 1.427)	10.793 (0.376, 1.573)	10.720 (0.425, 1.221)	1.0	10.910 (0.601, 1.428)	11.683 (0.720, 3.876)
FAM	11.117 (0.698, 1.770)	11.199 (0.676, 2.110)	11.018 (0.620, 1.672)	10.876 (0.376, 1.893)	10.793 (0.413, 1.503)	11.098 (0.700, 1.664)	1.0	11.849 (0.705, 4.521)
PLA	10.603 (0.272, 1.401)	10.646 (0.273, 1.620)	10.549 (0.233, 1.381)	10.470 (0.162, 1.342)	10.426 (0.171, 1.098)	10.594 (0.258, 1.389)	10.541 (0.221, 1.419)	1.0


*Each number is an odds ratio (=row/column), and 95% confidence interval. OME, omeprazole, 20 mg/day; PAN, pantoprazole, 40 mg/day; LAN, lansoprazole, 30 mg/day; RAB, rabeprazole, 20 mg/day; ILA, ilaprazole, 10 mg/day; RAN, ranitidine, 300 mg/day; FAM, famotidine, 40 mg/day; PLA, placebo. Green shading: no significant difference.*

**Table 3:** The incidence of adverse events for different intervention.

Adverse events	OME	PAN	LAN	RAB	ILA	RAN	FAM	PLA
Headache (%)	2.57	1.79	1.88	3.49	0.46	2.70	3.20	4.21
Diarrhea (%)	1.58	1.54	1.16	2.30	1.83	1.70	1.14	3.92
Nausea (%)	2.14	1.02	1.73	0.53	NR	0.88	1.90	3.92
Rash (%)	1.01	1.10	1.60	1.47	NR	0.76	0.92	NR
Vomiting (%)	0.25	NR	NR	0.53	2.02	0.57	1.11	3.92
Dizziness (%)	1.52	2.05	1.05	NR	NR	0.81	0.84	NR
Constipation (%)	0.18	0.80	2.21	NR	NR	0.63	1.32	NR
Myalgia	0.84	0.48	1.05	0.53	NR	0.59	NR	NR
Loss of appetite (%)	2.36	NR	1.82	NR	2.11	NR	1.17	NR
Insomnia (%)	1.09	2.50	1.82	NR	NR	NR	0.84	NR
Dry mouth (%)	2.37	2.17	0.80	NR	NR	NR	1.27	NR
Psychiatric disorder (%)	2.84	NR	0.42	NR	NR	1.85	1.41	NR
Cardiovascular disorder (%)	0.33	NR	0.57	0.53	NR	0.84	NR	NR
Asthenia (%)	2.77	NR	2.87	NR	NR	0.44	NR	NR
Somnolence (%)	1.12	NR	NR	NR	NR	1.22	0.78	NR
Abdominal distension (%)	1.58	NR	NR	NR	NR	1.91	2.20	NR
Abnormal liver function (%)	1.23	1.02	NR	NR	3.63	NR	NR	NR
Renal impairment (%)	0.19	NR	NR	NR	1.45	0.94	NR	NR


*NR, not reported. OME, omeprazole, 20 mg/day; PAN, pantoprazole, 40 mg/day; LAN, lansoprazole, 30 mg/day; RAB, rabeprazole, 20 mg/day; ILA, ilaprazole, 10 mg/day; RAN, ranitidine, 300 mg/day; FAM, famotidine, 40 mg/day; PLA, placebo.*

### Sensitivity Analyses

Sensitivity analyses comparing data from ITT populations to PP populations were presented in Table 1 and Supplementary Table S6, and analyses based on data from trials with lower risk of bias were shown in Supplementary Tables S7, S8, which showed similar results for both outcomes.

### Subgroup Analyses

Considering the impact of ethnicity on the results, we performed subgroup analyses in Chinese and non-Chinese participants, respectively. As shown in Supplementary Tables S9, S10, ILA tended to be more effective in improving 4-UHR in Chinese compared to non-Chinese participants. Chinese and non-Chinese subgroups showed similar results for incidence of AEs (Supplementary Tables S11, S12).

### Cost-Effectiveness Results

Table 4 and Supplementary Figure S6 presented the base-case results for a duration of 1 year: OME had the lowest expected total cost ($53023.30) for 10,000 Chinese patients with DU, followed by PAN, LAN, RAB, and ILA. ILA had the highest expected quality adjusted life years (QALYs) (8110.18), followed by RAB, PAN, LAN, and OME. OME was used as the baseline in calculating the ICERs of other strategies. The ICERs for PAN, LAN, RAB, and ILA relative to OME corresponded to $5134.67 per QALY, $17801.67 per QALY, $25488.31 per QALY, and $44572.22 per QALY, respectively. In the present cohorts, ILA was associated with the best efficacy with respect to incremental QALYs but it also had the highest costs. PAN, LAN, and RAB were also associated with greater efficacy but higher costs than OME. According to the threshold recommended by WHO, PAN was preferred based on its efficacy at an acceptable cost. Nevertheless, ILA was found not to be a strongly recommended treatment for patients in China, since the ICER corresponded to higher than $25683.33.

**Table 4:** Cost-effectiveness of PPIs for treatment of duodenal ulcer patients.

Treatment strategy	Cost (US$)	Incremental cost (US$)	QALYs	Incremental QALYs	ICER
OME	53023.30	NA	8077.61	NA	NA
PAN	126543.21	73519.91	8091.93	14.32	5134.67
LAN	304323.48	251300.18	8091.72	14.12	17801.67
RAB	430823.73	377800.43	8092.43	14.82	25488.31
ILA	1504703.48	1451680.18	8110.18	32.57	44572.22


*QALY, quality adjusted life year; ICER, incremental cost-effectiveness ratio, Calculated as the average cost per patient and the average number of QALYs per patient in this strategy minus those of the treatment of OME; OME, omeprazole, 20 mg/day; PAN, pantoprazole, 40 mg/day; LAN, lansoprazole, 30 mg/day; RAB, rabeprazole, 20 mg/day; ILA, ilaprazole, 10 mg/day; NA, not applicable.*

Probabilistic sensitivity analyses (Supplementary Table S13) with 1,000 Monte Carlo simulations revealed that PAN, LAN, RAB, and ILA had probabilities of 73.1% (Supplementary Figure S8), 60.6% (Supplementary Figure S9), 60.9% (Supplementary Figure S10), 15.2% (Supplementary Figure S11), respectively, of being cost-effective relative to OME under the threshold ($25683.33) currently accepted in China.

## Discussion

To our best knowledge, this is the first systematic review incorporating a network meta-analysis and cost-effectiveness analysis to compare PPIs for the initial non-eradication treatment of DU, and recommend a rank order based on efficacy, safety, and cost. Our study suggests that all the PPIs significantly improve the 4-UHR compared to H_2_RAs and PLA, while there is no significant difference for 4-UHR among PPIs. The incidences of AEs of PPIs, H_2_RAs, and PLA are similar during 4-week follow-up. PAN seems to be the most cost-effective choice in the initial non-eradication treatment of DU in China.

Most guidelines recommended that all patients with peptic ulcers should be tested for infection with Hp and treated (Malfertheiner et al., 2017). Nevertheless, an overview of systematic reviews and network meta-analysis (Xin et al., 2016) concluded that triple therapy with different antibiotics would influence the eradication rate which was associated with healing rate. In order to reduce the clinical heterogeneity caused by different antibiotics, this review evaluated the efficacy, safety, and cost-effectiveness of different PPIs in the non-eradication treatment of DU. At present, there are six PPIs (OME, PAN, LAN, RAB, ESO, and ILA) in the pharmaceutical market, but only five PPIs were included in this study. The main reason was that the ESO was more effective in the inhibition of gastric acid secretion (Beck, 2004; McKeage et al., 2008) and utilized more for the eradication of Hp, instead of non-eradication treatment of DU.

The subgroup analyses suggested that ILA obtained much better efficacy in Chinese rather than non-Chinese. The reason could be attributed to the fact that most RCTs including ILA were conducted in China, and one RCT (Chen et al., 2017) with high risk of bias reported extremely high 4-UHR of ILA in Chinese. After excluding that RCT, there was no significantly difference in the 4-UHR between ILA and other PPIs irrespective of Chinese or non-Chinese, which was consistent with the meta-analysis conducted by Ji et al. (2014).

A previous NMA (Hu et al., 2017) including 24 RCTs and 6,188 patients showed no significant difference for the efficacy and tolerance between the ordinary doses of different PPIs, which was mostly consistent with our study. However, we included more RCTs (62) and participants (10,339) to make the conclusion of NMA more robust. In addition, in order to perform the pharmacoeconomic analysis, our study only included the standard dose of PPIs rather than LAN (15 mg/day or 60 mg/day) or OME (40 mg/day).

The cost-effectiveness analysis indicated that ILA did not dominate OME, which was inconsistent with the previous study conducted by Xuan et al. (2016). This could be attributed to the different cost of OME applied in the model: The cost of OME in Xuan’s study was set as 16 yuan/day ($2.5456/day) exceeding the upper limit value in our study. The price of OME was reduced greatly because of greater competition and supply of OME in the domestic market. The data of drug cost in our study was from the National Health and Family Planning Commission of the People’s Republic of China and had better representativeness.

There are several limitations in this study. We only included RCTs in this review and were therefore underpowered to find rare AEs related to the medications, as the sample size was relatively small and the follow-up time was indeed short. On the other hand, some included RCTs, especially those from China had poor methodological quality, but results and interpretation did not change when these trials were excluded from the analyses. Due to few trials reporting the results of patients with CYP2C19 genotype, our study did not analyze this genotype stratification.

## Conclusion

This study suggests that the efficacy and tolerance of different PPIs are similar in the initial non-eradication treatment of DU, but PAN (40 mg/day) seems to be the most cost-effective choice in China. More RCTs are warranted to compare the efficacy, long term safety, and cost-effectiveness of different PPIs across different CYP2C19 genotypes.

## Author Contributions

JZ, LG, MH, YL, JX, DC, XL, WZ, and RH conceptualized and designed the experiments, critically revised the manuscript for important intellectual content, and approved the final version to be published including the authorship list. JZ, JX, and XL contributed to literature search and data collection. JZ and LG analyzed the statistical data. JZ, LG, and XL interpreted the data. JZ, LG, MH, YL, XL, and WZ drafted the manuscript.

## Conflict of Interest Statement

The authors declare that the research was conducted in the absence of any commercial or financial relationships that could be construed as a potential conflict of interest.

## Footnotes

1 http://www.nhfpc.gov.cn

2 http://data.stats.gov.cn/easyquery.htm?cn=C01&zb=A0201&sj=2016

## References

[B1] Ahmed W. Qureshi H. Zuberi S. J. Alam S. E. (1993). Omeprazole vs ranitidine in the healing of duodenal ulcer. *J. Pak. Med. Assoc.* 43 111–112. 8411611

[B2] Albaqawi A. S. B. El-Fetoh N. M. A. Alanazi R. F. A. Alanazi N. S. F. Alrayya S. E. Alanazi A. N. M. (2017). Profile of peptic ulcer disease and its risk factors in Arar, Northern Saudi Arabia. *Electron. Phys.* 9 5740–5745. 10.19082/5740 29403613 PMC5783122

[B3] Alcalá-Santaella R Guardia J. Pajares J. Pique J. Pita L. Alvárez E. (1989). A multicenter, randomized, double-blind study comparing a daily bedtime administration of famotidine and ranitidine in short-term treatment of active duodenal ulcer. *Hepatogastroenterology* 36 168–171. 2666293

[B4] Aro P. Storskrubb T. Ronkainen J. Bolling-Sternevald E. Engstrand L. Vieth M. (2006). Peptic ulcer disease in a general adult population: the Kalixanda study: a random population-based study. *Am. J. Epidemiol.* 163 1025–1034. 10.1093/aje/kwj129 16554343

[B5] Barbara L. Corinaldesi R. Bianchi Porro G. Lazzaroni M. Blasi A. Mangiameli A. (1985). Famotidine in the management of duodenal ulcer: experience in Italy. *Digestion* 32 24–31. 10.1159/000199258 2866133

[B6] Bardhan K. D. Bianchi Porro G Bose K. Daly M. Hinchliffe R. F. Jonsson E. (1986). A comparison of two different doses of omeprazole versus ranitidine in treatment of duodenal ulcers. *J. Clin. Gastroenterol.* 8 408–413. 10.1097/00004836-198608000-00005 3531313

[B7] Beck J. (2004). Efficacy of esomeprazole in patients with acid-peptic disorders. *Gastroenterol. Nurs.* 27 44-49. 10.1097/00001610-200403000-00002 15082946

[B8] Breiter J. R. Riff D. Humphries T. J. (2000). Rabeprazole is superior to ranitidine in the management of active duodenal ulcer disease: results of a double-blind, randomized North American study. *Am. J. Gastroenterol.* 95 936–942. 10.1111/j.1572-0241.2000.01933.x 10763941

[B9] Chang F. Y. Chiang C. Y. Tam T. N. Ng W. W. Lee S. D. (1995). Comparison of lansoprazole and omeprazole in the short-term management of duodenal ulcers in Taiwan. *J. Gastroenterol. Hepatol.* 10 595–601. 10.1111/j.1440-1746.1995.tb01352.x 8963037

[B10] Chelvam P. Goh K. L. Leong Y. P. Leela M. P. Yin T. P. Ahmad H. (1989). Omeprazole compared with ranitidine once daily in the treatment of duodenal ulcer. *J. Gastroenterol.* 4 53–61. 2491362

[B11] Chen C. Kuang C. Q. Lai J. Y. (2017). Comparative study of ilaprazole versus omeprazole in the treatment of duodenal ulcer. *J. North Phar.* 14:38. 23158273

[B12] Chen S. (2017). Analysis on treatment method and effect of duodenal ulcer in department of gastroenterology. *Chin. J. Mod. Drug Appl.* 11 22–24.

[B13] Clinical Study Group of Pantoprazole in Shanghai (2001). Pantoprazole in the treatment of active peptic ulcer. *Chin. J. Dig.* 21 22–24.

[B14] Cloud M. L. Enas N. Humphries T. J. Bassion S. (1998). Rabeprazole in treatment of acid peptic diseases: results of three placebo-controlled dose-response clinical trials in duodenal ulcer, gastric ulcer, and gastroesophageal reflux disease (GERD), the rabeprazole study group. *Dig. Dis. Sci.* 43 993–1000. 10.1023/A:1018822532736 9590413

[B15] Cremer M. Lambert R. Lamers C. B. Delle Fave G. Maier C. (1994). Improved duodenal ulcer healing with pantoprazole compared with ranitidine: a multicentre study. *Eur. J. Gastroen Hepat.* 6 739–743. 10.1097/00042737-199408000-00018 7781461

[B16] Cremer M. Lambert R. Lamers C. B. Delle Fave G. Maier C. (1995). A double-blind study of pantoprazole and ranitidine in treatment of acute duodenal ulcer. A multicenter trial. European Pantoprazole Study Group. *Dig. Dis. Sci.* 40 1360–1364. 10.1007/BF02065552 7781461

[B17] Dekkers C. P. Beker J. A. Thjodleifsson B. Gabryelewicz A. Bell N. E. Humphries T. J. (1999). Comparison of rabeprazole 20mg versus omeprazole 20mg in the treatment of active duodenal ulcer: a European multicenter study. *Aliment. Pharmacol. Ther.* 13 179–186. 10.1046/j.1365-2036.1999.00449.x 10102948

[B18] Delle Fave G Annibale B. Franceschi M. Quatrini M. Cassetta M. R. Torsoli A. (1992). Omeprazole versus famotidine in the short-term treatment of duodenal ulcer disease. *Aliment. Pharmacol. Ther.* 6 469–478. 10.1111/j.1365-2036.1992.tb00560.x 1420739

[B19] DerSimonian R. Laird N. (1986). Meta-analysis in clinical trials. *Control Clin. Trials* 7 177–188. 10.1016/0197-2456(86)90046-2 3802833

[B20] Dobrilla G. De Pretis G Piazzi L. Boero A. Camarri E. Crespi M. (1987). Comparison of once-daily bedtime administration of famotidine and ranitidine in the short-term treatment of duodenal ulcer a multicenter, double-blind, controlled study. *Scand. J. Gastroenterol. Suppl.* 134 21–28. 10.3109/00365528709090136 2889255

[B21] Editorial Board of Chinese Jounarl of Digestion (2016). Diagnosis and treatment of peptic ulcer (2016, Xian). *Chin. J. Dig.* 36 508–513.

[B22] Ekström P. Carling L. Unge P. Anker-Hansen O. Sjöstedt S. Sellström H. (1995). Lansoprazole versus omeprazole in active duodenal ulcer, A double-blind, randomized, comparative study. *Scand. J. Gastroenterol.* 30 210–215. 10.3109/00365529509093265 7770708

[B23] Ford A. C. Gurusamy K. S. Delaney B. Forman D. Moayyedi P. (2016). Eradication therapy for peptic ulcer disease in *Helicobacter pylori*-positive people. *Cochrane Database Syst. Rev.* 4:CD003840. 10.1002/14651858.CD003840.pub5 27092708 PMC7163278

[B24] Gao W. Cheng H. Hu F. L. Lü N. H. Xie Y. Sheng J. Q. (2017). Comparative study of ilaprazole versus omeprazole in the treatment of duodenal ulcer. *J. N. Pharmacy* 14:38. 23158273

[B25] Ghosh C. K. Khan M. R. Alam F. Shil B. C. Kabir M. S. Mahmuduzzaman M. (2017). Peptic ulcer disease in Bangladesh: a multi-centre study. *Mymensingh Med. J.* 26 141–144. 28260768

[B26] Graham D. Y. McCullough A. Sklar M. Sontag S. J. Roufail W. M. Stone R. C. (1990). Omeprazole versus placebo in duodenal ulcer healing. The United States experience. *Dig. Dis. Sci.* 35 66–72. 10.1007/BF01537225 2403908

[B27] Groeneveld P. W. Lieu T. A. Fendrick A. M. Hurley L. B. Ackerson L. M. Levin T. R. (2001). Quality of life measurement clarifies the cost-effectiveness of *Helicobacter pylori* eradication in peptic ulcer disease and uninvestigated dyspepsia. *Am. J. Gastroenterol.* 96 338–347. 10.1016/S0002-9270(00)02309-1 11232673

[B28] Gu W. (2005). Comparative study of omeprazole versus famotidine in the treatment of duodenal ulcers. *Cent. Plains Med. J.* 32 50–51.

[B29] Hallerback B. Glise H. Svedlund J. Agreus L. Gäcke-Herbst R. Engstrand C. (1998). Quality of life in duodenal ulcer treatment: a comparison of omeprazole and ranitidine in acute and intermittent treatment. *Psychol. Health Med.* 3 417–427. 10.1080/13548509808400615

[B30] Hawkey C. J. Long R. G. Bardhan K. D. Wormsley K. G. Cochran K. M. Christian J. (1993). Improved symptom relief and duodenal ulcer healing with lansoprazole, a new proton pump inhibitor, compared with ranitidine. *Gut* 34 1458–1462. 10.1136/gut.34.10.1458 8244121 PMC1374562

[B31] Higgins J. Green S. (2011). *The Cochrane Collaboration. Cochrane Handbook for Systematic Reviews of Interventions Version 5.1.0.* Available at: http://handbook.cochrane.org/

[B32] Ho K. Y. Kuan A. Zaño F. Goh K. L. Mahachai V. Kim D. Y. (2009). Randomized, parallel, double-blind comparison of the ulcer-healing effects of ilaprazole and omeprazole in the treatment of gastric and duodenal ulcers. *J. Gastroenterol.* 44 697–707. 10.1007/s00535-009-0072-4 19434360

[B33] Hotz J. Kleinert R. Grymbowski T. Hennig U. Schwarz J. A. (1992). Lansoprazole versus famotidine: efficacy and tolerance in the acute management of duodenal ulceration. *Aliment. Pharmacol. Ther.* 6 87–95. 10.1111/j.1365-2036.1992.tb00548.x 1543819

[B34] Hu Z. H. Shi A. M. Hu D. M. Bao J. J. (2017). Efficacy of proton pump inhibitors for patients with duodenal ulcers: a pairwise and network meta-analysis of randomized controlled trials. *Saudi J. Gastroenterol.* 23 11–19. 10.4103/1319-3767.199117 28139495 PMC5329971

[B35] Hu N. Z. (2001). *A Clinical Study and Evaluation of 5-aminosalicylic Acid Enteric-Coated Tablets, Mosapride and Rabeprazole.* The Master Degree Thesis, Medical University of Anhui, 7–33.

[B36] Huang J. R. (2010). Observation of the efficacy of pantoprazole versus omeprazole in the treatment of duodenal ulcers. *Chin. J. Mod. Drug Appl.* 4 160–161.

[B37] Hui W. M. Lam S. K. Lau W. Y. Branicki F. J. Lok A. S. Ng M. M. (1989). Omeprazole and ranitidine in duodenal ulcer healing and subsequent relapse: a randomized double-blind study with weekly endoscopic assessment. *J. Gastroenterol. Hepatol.* 4 35–43. 2491360

[B38] Hutubessy R. Dan C. Edejer T. T. (2003). Generalized cost-effectiveness analysis for national-level priority-setting in the health sector. *Cost Eff. Resour. Alloc.* 1 1–13. 10.1186/1478-7547-1-8 14687420 PMC320499

[B39] Ji X. Q. Du J. F. Chen G. Chen G. Yu B. (2014). Efficacy of ilaprazole in the treatment of duodenal ulcers: a meta-analysis. *World J. Gastroenterol.* 20 5119–5123. 10.3748/wjg.v20.i17.5119 24803828 PMC4009550

[B40] Judmaier G. Koelz H. R. (1994). Comparison of pantoprazole and ranitidine in the treatment of acute duodenal ulcer. Pantoprazole-Duodenal Ulcer-Study Group. *Aliment Pharmacol. Ther.* 8 81–86. 10.1111/j.1365-2036.1994.tb00163.x 8186350

[B41] Kumar T. R. Naidu M. U. Shobha J. C. Reddy D. N. Subhash S. Chaubal C. (1992). Comparative study of omeprazole and famotidine in the treatment of duodenal ulcer. *Indian J. Gastroenterol.* 11 73–75. 1428035

[B42] Lanza F. Goff J. Scowcroft C. Jennings D. Greski-Rose P. (1994). Double-blind comparison of lansoprazole, ranitidine, and placebo in the treatment of acute duodenal ulcer. *Am. J. Gastroenterol.* 89 1191–1200. 8053433

[B43] Leodolter A. Kulig M. Brasch H. Meyer-Sabellek W. Willich S. N. Malfertheiner P. (2001). A meta-analysis comparing eradication, healing and relapse rates in patients with *Helicobacter pylori*-associated gastric or duodenal ulcer. *Aliment Pharmacol. Ther.* 15 1949–1958. 10.1046/j.1365-2036.2001.01109.x 11736726

[B44] Li D. F. Zhang L. H. Wang Y. F. Kusaka S. Asagoe K. Dekigai H. (1994). The effect of omeprazole in the elder patients with peptic ulcer. *Chin. J. Gerontol.* 14 273–275.

[B45] Li N. Gu K. H. Chi C. Zhou J. F. (2001). Contrastive study of the healing and relapse rates in duodenal ulcer disease treated with omeprazole and ranitidine. *Clin. Med. China* 17 108–109.

[B46] Li Y. Li W. H. (2010). Pantoprazole for active peptic ulcer. *China For. Med. Treat.* 3 22–23.

[B47] Li Z. Zou D. Ma X. Chen J. Shi X. Gong Y. (2010). Epidemiology of peptic ulcer disease: endoscopic results of the systematic investigation of gastrointestinal disease in China. *Am. J. Gastroenterol.* 105 2570–2577. 10.1038/ajg.2010.324 20736940

[B48] Liao Z. C. (2015). Analysis of the clinical effect of ilaprazole in the treatment of duodenal ulcer. *Jilin Med. J.* 36 3581–3582. 27223846

[B49] Lin K. J. Garcia Rodriguez L. A. Hernandez-Diaz S. (2011). Systematic review of peptic ulcer disease incidence rates: do studies without validation provide reliable estimates? *Pharmacoepidemiol. Drug Saf.* 20 718–728. 10.1002/pds.2153 21626606

[B50] Liu B. Cheng T. (2011). Observation of the efficacy of pantoprazole in the treatment of duodenal ulcers. *Chin. J. Clin. Gastroenterol.* 23 106–107.

[B51] Londong W. Barth H. Dammann H. G. Hengels K. J. Kleinert R. Müller P. (1991). Dose-related healing of duodenal ulcer with the proton pump inhibitor lansoprazole. *Aliment. Pharmacol. Ther.* 5 245–254. 10.1111/j.1365-2036.1991.tb00025.x 1888824

[B52] Lysy J. Karmeli F. Wengrower D. Rachmilewitz D. (1992). Effect of duodenal ulcer healing induced by omeprazole and ranitidine on the generation of gastroduodenal eicosanoids, platelet-activating factor, pepsinogen A, and gastrin in duodenal ulcer patients. *Scand. J. Gastroenterol.* 27 13–19. 10.3109/00365529209011159 1736336

[B53] Malfertheiner P. Megraud F. O’Morain C. A. Gisbert J. P. Kuipers E. J. Axon A. T. (2017). Management of *Helicobacter pylori* infection-the maastricht V/florence consensus report. *Gut* 66 6–30. 10.1136/gutjnl-2016-312288 27707777

[B54] Marks I. N. Danilewitz M. D. Garisch J. A. (1991). A comparison of omeprazole and ranitidine for duodenal ulcer in South African patients. A multiracial study. *Dig. Dis. Sci.* 36 1395–1400. 10.1007/BF01296805 1914761

[B55] Marks I. N. Wright J. P. (1987). Comparison of famotidine 40mg with ranitidine 300mg at night in short-term duodenal ulcer healing: a south African multicenter study. *S. Afr. Med. J.* 72 18–20. 2885928

[B56] Mccullough A. J. (1986). A multicenter, randomized, double-blind study comparing famotidine with ranitidine in the treatment of active duodenal ulcer disease. *Am. J. Med.* 81 17–24. 10.1016/0002-9343(86)90596-6 2877570

[B57] McFarland R. J. Bateson M. C. Green J. R. O’Donoghue D. P. Dronfield M. W. Keeling P. W. (1990). Omeprazole provides quicker symptom relief and duodenal ulcer healing than ranitidine. *Gastroenterology* 98 278–283. 10.1016/0016-5085(90)90815-I 2403952

[B58] McKeage K. Blick S. K. Croxtall J. D. Lyseng-Williamson K. A. Keating G. M. (2008). Esomeprazole: a review of its use in the management of gastric acid-related diseases in adults. *Drugs* 68 1571-1607. 10.2165/00003495-200868110-00009 18627213

[B59] Meneghelli U. G. Zaterka S. de Paula Castro L. Malafaia O. Lyra L. G. (2000). Pantoprazole versus ranitidine in the treatment of duodenal ulcer: a multicenter study in Brazil. *Am. J. Gastroenterol.* 95 62–66. 10.1111/j.1572-0241.2000.01745.x 10638560

[B60] Misra S. C. Dasarathy S. Sharma M. P. (1993). Omeprazole versus famotidine in the healing and relapse of duodenal ulcer. *Aliment. Pharmacol. Ther.* 7 443–449. 10.1111/j.1365-2036.1993.tb00118.x 8218758

[B61] Mulder C. J. Tijtgat G. N. Cluysenaer O. J. Nicolai J. J. Meyer W. W. Hazenberg B. P. (1989). Omeprazole (20mg o.m*.)* versus ranitidine *(*150mg b.d.) in duodenal ulcer healing and pain relief. *Aliment. Pharmacol. Ther.* 3 445–451. 10.1111/j.1365-2036.1989.tb00235.x 2518857

[B62] Pei Y. Wang H. J. Yu Z. L. Wang B. E. Dai X. Z. Dong E. Y. (2000). Comparative study of pantoprazole versus omeprazole in patients with duodenal ulcer. *Chin. New Drugs J.* 9 117–119.

[B63] Rehner M. Rohner H. G. Schepp W. (1995). Comparison of pantoprazole versus omeprazole in the treatment of acute duodenal ulceration: a multicenter study. *Aliment. Pharmacol. Ther.* 9 411–416. 10.1111/j.1365-2036.1995.tb00399.x 8527617

[B64] Rensburg V. Christoffel J. Eden V. (1994). Improved duodenal ulcer healing with pantoprazole compared with ranitidine: A multicentre study. *Eur. J. Gastroen. Hepat.* 6 739–743. 7781461

[B65] Satoh K. Yoshino J. Akamatsu T. Itoh T. Kato M. Kamada T. (2016). Evidence-based clinical practice guidelines for peptic ulcer disease 2015. *J. Gastroenterol.* 51 177–194. 10.1007/s00535-016-1166-4 26879862

[B66] Schepp W. Classen M. (1995). Pantoprazole and ranitidine in the treatment of acute duodenal ulcer. A multicentre study. *Scand. J. Gastroenterol.* 30 511–514. 10.3109/00365529509089781 7569755

[B67] Shin J. M. Cho Y. M. Sachs G. (2004). Chemistry of covalent inhibition of the gastric (H+.K+)-ATPase by proton pump inhibitors. *J. Am. Chem. Soc.* 126 7800–7811. 10.1021/ja049607w 15212527

[B68] Simon B. Dammann H. G. Jakob G. Miederer S. E. Müller P. Ottenjann R. (1985). Famotidine versus ranitidine for the short-term treatment of duodenal ulcer. *Digestion* 32 32–37. 10.1159/000199259 2866134

[B69] Sun S. Chen J. Johannesson M. Kind P. Xu L. Zhang Y. (2011). Population health status in China: EQ-5D results, by age, sex and socio-economic status, from the National Health Services Survey 2008. *Qual Life Res.* 20 309–320. 10.1007/s11136-010-9762-x 21042861 PMC3052443

[B70] Tang S. H. Hu W. (2001). Effect of pantoprazole in the treatment of peptic ulcer. *Herald Med.* 20 431–432.

[B71] Tao Z. H. Yin H. W. (1993). Study on a new and China made anti-ulcer drug omeprazole. *Chin. N Drugs J.* 2 1–3.

[B72] Valenzuela J. E. Berlin R. G. Snape W. J. Johnson T. L. Hirschowitz B. I. Colon-Pagan J. (1991). U.S. experience with omeprazole in duodenal ulcer. Multicenter double-blind comparative study with ranitidine. The Omeprazole DU Comparative Study Group. *Dig. Dis. Sci.* 36 761–768. 10.1007/BF01311234 2032518

[B73] van Valkenhoef G. Dias S. Ades A. E. Welton N. J. (2016). Automated generation of node-splitting models for assessment of inconsistency in network meta-analysis. *Res. Synth. Methods* 7 80–93. 10.1002/jrsm.1167 26461181 PMC5057346

[B74] van Valkenhoef G. Lu G. de Brock B. Hillege H. Ades A. E. Welton N. J. (2012). Automating network meta-analysis. *Res. Synth. Methods* 3 285–299. 10.1002/jrsm.1054 26053422

[B75] Wang C. Y. Wang T. H. Lai K. H. Siauw C. P. Chen P. C. Yang K. C. (1992). Double-blind comparison of omeprazole 20 mg OM and ranitidine 300 mg NOCTE in duodenal ulcer: a Taiwan multi-centre study. *J. Gastroenterol. Hepatol.* 7 572–576. 10.1111/j.1440-1746.1992.tb01488.x 1486186

[B76] Wang L. Zhou L. Hu H. Lin S. Xia J. (2012). Ilaprazole for the treatment of duodenal ulcer: a randomized, double-blind and controlled phase III trial. *Curr. Med. Res. Opin.* 28 101–109. 10.1185/03007995.2011.639353 22070512

[B77] Wang L. Zhou L. Lin S. Hu H. Xia J. (2011). A new PPI, ilaprazole compared with omeprazole in the treatment of duodenal ulcer: a randomized double-blind multicenter trial. *J. Clin. Gastroenterol.* 45 322–329. 10.1097/MCG.0b013e3181e88515 20679904

[B78] Wang P. Chen W. (2013). Observation of the effect of ilaprazole in the elder patients with duodenal ulcer. *Mod. J. Integr. Tradit. Chin. Western Med.* 22 763–764.

[B79] Wang X. P. Liu H. Y. Wang J. Y. Jiang S. H. Yuan Y. Z. Xu G. M. (2000). Efficacy of proton pump inhibitor on duodenal ulcer: comparison study between pantoprazole and lansoprazole. *Chin. New Drugs J.* 9 120–122.

[B80] Wolfe M. M. Sachs G. (2000). Acid suppression: optimizing therapy for gastroduodenal ulcer healing, gastroesophageal reflux disease, and stress-related erosive syndrome. *Gastroenterology* 118 S9–S31. 10.1016/S0016-5085(00)70004-7 10868896

[B81] Xiao Q. (1997). Lansoprazole for the treatment of peptic ulcer. *J. Yunyang Med. Coll.* 16 36–38.

[B82] Xin Y. Manson J. Govan L. Harbour R. Bennison J. Watson E. (2016). Pharmacological regimens for eradication of *Helicobacter pylori*: an overview of systematic reviews and network meta-analysis. *BMC Gastroenterol.* 16:80. 10.1186/s12876-016-0491-7 27460211 PMC4962503

[B83] Xu J. Y. (2001). *Clinical Study of Rabeprazole in the Treatment of Peptic Ulcer.* The Master Degree Thesis, Sichuan University, Sichuan 1–21.

[B84] Xu Y. H. (2006). Observation of the clinical effect of rabeprazole in the treatment of duodenal ulcers. *China Med. Herald.* 3:94. 10.1186/1749-8546-1-2 17302965 PMC1761146

[B85] Xuan J. W. Song R. L. Xu G. X. Lu W. Q. Lu Y. J. Liu Z. (2016). Modeling the cost-effectiveness of ilaprazole versus omeprazole for the treatment of newly diagnosed duodenal ulcer patients in China. *J. Med. Econ.* 19 1056–1060. 10.1080/13696998.2016.1194277 27223846

[B86] You Q. X. Yang J. L. Jiang S. H. (2006). Observation of the efficacy of rabeprazole in the treatment of duodenal ulcers. *People Mil Surg.* 49 77–78.

[B87] Zagari R. M. Law G. R. Fuccio L. Pozzato P. Forman D. Bazzoli F. (2010). Dyspeptic symptoms and endoscopic findings in the community: the Loiano-Monghidoro study. *Am. J. Gastroenterol.* 105 565–571. 10.1038/ajg.2009.706 20010920

[B88] Zaterka S. Massuda H. Chinzon D. Eisig J. N. Miszputen S. Kendo M. (1993). Treatment of duodenal ulcer with omeprazole or ranitidine in a Brazilian population: a multicenter double-blind, parallel group study. *Am. J. Gastroenterol.* 88 397–401. 8438847

[B89] Zhao L. R. Sun Q. W. Wang Y. Y. (2013). Comparison of pantoprazole versus omeprazole in the treatment of duodenal ulcers. *Chin. For. Med. Res.* 11 45–46.

[B90] Zou D. Q. (2012). Pantoprazole for 20 patients with duodenal ulcer. *Chin. Med. Modern Dist. Educ. China* 10:63.

