# Supplementary material for: Standard-Dose Proton Pump Inhibitors in the Initial Non-eradication Treatment of Duodenal Ulcer: Systematic Review, Network Meta-Analysis, and Cost-Effectiveness Analysis
Source: Front Pharmacol. 2019 Jan 7;9:1512. doi: 10.3389/fphar.2018.01512 (PMC6330312; doi:10.3389/fphar.2018.01512)
Supplement: Supplementary file 1 [file Data_Sheet_1.doc]

**Table S1. PRISMA NMA Checklist of Items to Include When Reporting A Systematic Review Involving a Network Meta-analysis**

| **Section/Topic** | **Item #** | **Checklist Item** | **Reported on Page #** |
| --- | --- | --- | --- |
| **TITLE** |  |  |  |
| Title | 1 | Identify the report as a systematic review *incorporating a network meta-analysis (or related form of meta-analysis).* | Page 1 |
|  |  |  |  |
| **ABSTRACT** |  |  |  |
| Structured summary | 2 | Provide a structured summary including, as applicable:  **Background:** main objectives  **Methods:** data sources; study eligibility criteria, participants, and interventions; study appraisal; and *synthesis methods, such as network meta-analysis.*  **Results:** number of studies and participants identified; summary estimates with corresponding confidence/credible intervals; *treatment rankings may also be discussed. Authors may choose to summarize pairwise comparisons against a chosen treatment included in their analyses for brevity.*  **Discussion/Conclusions:** limitations; conclusions and implications of findings.  **Other:** primary source of funding; systematic review registration number with registry name. | Page 1 to Page 2 |
|  |  |  |  |
| **INTRODUCTION** |  |  |  |
| Rationale | 3 | Describe the rationale for the review in the context of what is already known*, including mention of why a network meta-analysis has been conducted.* | Page 2 |
| Objectives | 4 | Provide an explicit statement of questions being addressed, with reference to participants, interventions, comparisons, outcomes, and study design (PICOS). | Page 3 |
|  |  |  |  |
| **METHODS** |  |  |  |
| Protocol and registration | 5 | Indicate whether a review protocol exists and if and where it can be accessed (e.g., Web address); and, if available, provide registration information, including registration number. | PROSPERO, CRD42017079704 |
| Eligibility criteria | 6 | Specify study characteristics (e.g., PICOS, length of follow-up) and report characteristics (e.g., years considered, language, publication status) used as criteria for eligibility, giving rationale. *Clearly describe eligible treatments included in the treatment network, and note whether any have been clustered or merged into the same node (with justification).* | Page 4 |
| Information sources | 7 | Describe all information sources (e.g., databases with dates of coverage, contact with study authors to identify additional studies) in the search and date last searched. | Page 3 |
| Search | 8 | Present full electronic search strategy for at least one database, including any limits used, such that it could be repeated. | Table S3 |
| Study selection | 9 | State the process for selecting studies (i.e., screening, eligibility, included in systematic review, and, if applicable, included in the meta-analysis). | Page 4 to Page 5 |
| Data collection process | 10 | Describe method of data extraction from reports (e.g., piloted forms, independently, in duplicate) and any processes for obtaining and confirming data from investigators. | Page 4 to Page 5 |
| Data items | 11 | List and define all variables for which data were sought (e.g., PICOS, funding sources) and any assumptions and simplifications made. | Page 4 to Page 5 |
| **Geometry of the network** | **S1** | Describe methods used to explore the geometry of the treatment network under study and potential biases related to it. This should include how the evidence base has been graphically summarized for presentation, and what characteristics were compiled and used to describe the evidence base to readers. | Page 5 |
| Risk of bias within individual studies | 12 | Describe methods used for assessing risk of bias of individual studies (including specification of whether this was done at the study or outcome level), and how this information is to be used in any data synthesis. | Page 5 |
| Summary measures | 13 | State the principal summary measures (e.g., risk ratio, difference in means). *Also describe the use of additional summary measures assessed, such as treatment rankings and surface under the cumulative ranking curve (SUCRA) values, as well as modified approaches used to present summary findings from meta-analyses.* | Page 5 to Page 6 |
| Planned methods of analysis | 14 | Describe the methods of handling data and combining results of studies for each network meta-analysis. This should include, but not be limited to:   - *Handling of multi-arm trials;* - *Selection of variance structure;* - *Selection of prior distributions in Bayesian analyses; and* - *Assessment of model fit.* | Page 5 to Page 6 |
| **Assessment of Inconsistency** | **S2** | Describe the statistical methods used to evaluate the agreement of direct and indirect evidence in the treatment network(s) studied. Describe efforts taken to address its presence when found. | Page 5 |
| Risk of bias across studies | 15 | Specify any assessment of risk of bias that may affect the cumulative evidence (e.g., publication bias, selective reporting within studies). | Page 5 |
| Additional analyses | 16 | Describe methods of additional analyses if done, indicating which were pre-specified. This may include, but not be limited to, the following:   - Sensitivity or subgroup analyses; - Meta-regression analyses; - *Alternative formulations of the treatment network; and* - *Use of alternative prior distributions for Bayesian analyses (if applicable).* | Page 6 |
|  |  |  |  |
| **RESULTS†** |  |  |  |
| Study selection | 17 | Give numbers of studies screened, assessed for eligibility, and included in the review, with reasons for exclusions at each stage, ideally with a flow diagram. | Page 7 and Figure 1 |
| **Presentation of network structure** | **S3** | Provide a network graph of the included studies to enable visualization of the geometry of the treatment network. | Figure 2(a) and Figure 2(b) |
| **Summary of network geometry** | **S4** | Provide a brief overview of characteristics of the treatment network. This may include commentary on the abundance of trials and randomized patients for the different interventions and pairwise comparisons in the network, gaps of evidence in the treatment network, and potential biases reflected by the network structure. | Page 7 |
| Study characteristics | 18 | For each study, present characteristics for which data were extracted (e.g., study size, PICOS, follow-up period) and provide the citations. | Page 7 and Table S4 |
| Risk of bias within studies | 19 | Present data on risk of bias of each study and, if available, any outcome level assessment. | Page 8 and Figure S1 |
| Results of individual studies | 20 | For all outcomes considered (benefits or harms), present, for each study: 1) simple summary data for each intervention group, and 2) effect estimates and confidence intervals. *Modified approaches may be needed to deal with information from larger networks.* | Figure S2 and Figure S4 |
| Synthesis of results | 21 | Present results of each meta-analysis done, including confidence/credible intervals. *In larger networks, authors may focus on comparisons versus a particular comparator (e.g. placebo or standard care), with full findings presented in an appendix. League tables and forest plots may be considered to summarize pairwise comparisons.* If additional summary measures were explored (such as treatment rankings), these should also be presented. | Page 8 to Page 10.  Table 2 to Table 3.  Table S5 to Table S12.  Figure S2 to Figure S5 |
| **Exploration for inconsistency** | **S5** | Describe results from investigations of inconsistency. This may include such information as measures of model fit to compare consistency and inconsistency models, *P* values from statistical tests, or summary of inconsistency estimates from different parts of the treatment network. | Page 8 to Page 9.  Figure S3 and Figure S5 |
| Risk of bias across studies | 22 | Present results of any assessment of risk of bias across studies for the evidence base being studied. | Page 8 |
| Results of additional analyses | 23 | Give results of additional analyses, if done (e.g., sensitivity or subgroup analyses, meta-regression analyses*, alternative network geometries studied, alternative choice of prior distributions for Bayesian analyses,* and so forth). | Page 9 to Page 10.  Table S6 to Table S12. |
|  |  |  |  |
| **DISCUSSION** |  |  |  |
| Summary of evidence | 24 | Summarize the main findings, including the strength of evidence for each main outcome; consider their relevance to key groups (e.g., healthcare providers, users, and policy-makers). | Page 10 to Page 11 |
| Limitations | 25 | Discuss limitations at study and outcome level (e.g., risk of bias), and at review level (e.g., incomplete retrieval of identified research, reporting bias). *Comment on the validity of the assumptions, such as transitivity and consistency. Comment on any concerns regarding network geometry (e.g., avoidance of certain comparisons).* | Page 12 |
| Conclusions | 26 | Provide a general interpretation of the results in the context of other evidence, and implications for future research. | Page 12 |
|  |  |  |  |
| **FUNDING** |  |  |  |
| Funding | 27 | Describe sources of funding for the systematic review and other support (e.g., supply of data); role of funders for the systematic review. This should also include information regarding whether funding has been received from manufacturers of treatments in the network and/or whether some of the authors are content experts with professional conflicts of interest that could affect use of treatments in the network. | None |

PICOS = population, intervention, comparators, outcomes, study design.

* Text in italics indicateS wording specific to reporting of network meta-analyses that has been added to guidance from the PRISMA statement.

† Authors may wish to plan for use of appendices to present all relevant information in full detail for items in this section.

**Table S3. Searching Strategy**

| **Database** | **Search Strategy** | **Results** |
| --- | --- | --- |
| PUBMED | #1 ("Duodenal Ulcer"[Mesh]) OR "duodenal ulcer"#2 ("Proton Pump Inhibitors"[Mesh]) OR "Proton Pump Inhibitors"#3 ("Omeprazole"[Mesh]) OR omeprazole#4 ("Rabeprazole"[Mesh]) OR rabeprazole#5 ("Lansoprazole"[Mesh]) OR lansoprazole#6 ("Esomeprazole"[Mesh]) OR esomeprazole#7 ("pantoprazole" [Supplementary Concept]) OR pantoprazole#8 ("ilaprazole" [Supplementary Concept]) OR ilaprazole#9 #2~#8 OR#10 #1 AND #9 #11 ("Famotidine"[Mesh]) OR Famotidine  #12 ("Ranitidine"[Mesh]) OR Ranitidine  #13 #1 AND #11 AND #12  #10 OR #13 | 1,770 |
| EMBASE | #1 'duodenum ulcer'/exp OR 'duodenum ulcer'  #2 'proton pump inhibitors' OR 'proton pump inhibitor'/exp  #3 'omeprazole'/exp OR omeprazole  #4 'rabeprazole'/exp OR rabeprazole  #5 'esomeprazole'/exp OR esomeprazole  #6 'lansoprazole'/exp OR lansoprazole  #7 'pantoprazole'/exp OR pantoprazole  #8 'ilaprazole'/exp OR ilaprazole  #9 #2~#8 OR  #10 #1 AND #9  #11 'ranitidine'/exp OR ranitidine  #12 'famotidine'/exp OR famotidine  #13 #1 AND #11 AND #12  #14 #10 OR #13 | 3,984 |
| COCHRANE | #1 "Duodenal Ulcer":ti,ab,kw or "Duodenal Ulcer" (Word variations have been searched)  #2 MeSH descriptor: [Duodenal Ulcer] explode all trees  #3 #1 OR #2 #4 "Proton Pump Inhibitors":ti,ab,kw or "Proton Pump Inhibitors" (Word variations have been searched)#5 MeSH descriptor: [Proton Pump Inhibitors] explode all trees#6 "omeprazole":ti,ab,kw or "omeprazole" (Word variations have been searched)#7 MeSH descriptor: [Omeprazole] explode all trees#8 "rabeprazole":ti,ab,kw or "rabeprazole" (Word variations have been searched)#9 MeSH descriptor: [Rabeprazole] explode all trees#10 "lansoprazole":ti,ab,kw or "lansoprazole" (Word variations have been searched)#11 MeSH descriptor: [Lansoprazole] explode all trees#12 "esomeprazole":ti,ab,kw or "esomeprazole" (Word variations have been searched)#13 MeSH descriptor: [Esomeprazole] explode all trees#14 "pantoprazole":ti,ab,kw or "pantoprazole" (Word variations have been searched)#15 ilaprazole:ti,ab,kw or ilaprazole (Word variations have been searched)#16 #4~#15#17 #3 and #16#18 "ranitidine":ti,ab,kw or "ranitidine" (Word variations have been searched)#19 MeSH descriptor: [Ranitidine] explode all trees#20 "famotidine":ti,ab,kw or "famotidine" (Word variations have been searched)#21 MeSH descriptor: [Famotidine] explode all trees#22 #18 or #19#23 #20 or #21#24 #3 and #22 and #23#25 #17 or #24 | 1,023 |

**Table S4.** Characteristics of the included studies (N=62)

| ***Study***  ***ID*** | ***Study Design*** | ***Country***  ***/Region*** | ***Population***  ***(n)*** | ***Treatment***  ***(n)*** | ***Baseline*** | | | | ***Duration of treatment***  ***(weeks)*** | ***Outcome*** |
| --- | --- | --- | --- | --- | --- | --- | --- | --- | --- | --- |
|  |  |  |  |  | ***Male***  ***(n)*** | ***Age***  ***(years)*** | ***Smokers***  ***(n)*** | ***Alcohol***  ***(n)*** |  |  |
| Li Y  2010 | RCT | China | DU (57) | OME (29)  PAN (28) | *NA* | 16-70 | *NA* | *NA* | 4 | ①② |
| SHPT group  2001 | RCT | China | DU (74) | OME (38)  PAN (36) | *NA* | 47.1±13.8  45.4±11.7 | *NA* | *NA* | 4 | ①② |
| Zhao LR  2013 | RCT | China | DU (76) | OME (36)  PAN (40) | 54 | 29-69, 47.4  30-67, 44.8 | *NA* | *NA* | 4 | ① |
| Pei Y  2000 | RCT | China | DU (120) | OME (60)  PAN (60) | 90 | 18-75 | *NA* | *NA* | 4 | ①② |
| Huang JR  2010 | RCT | China | DU (120) | OME (60)  PAN (60) | 72 | 20-65, 36.5±10.2 | *NA* | *NA* | 4 | ①② |
| Zou DQ  2012 | RCT | China | DU (40) | OME (20)  PAN (20) | 26 | 19-62 | *NA* | *NA* | 4 | ①② |
| Liu B  2011 | RCT | China | DU (118) | OME (58)  PAN (60) | 80 | 47.1±13.8  45.4±11.7 | *NA* | *NA* | 4 | ①② |
| Chen S  2017 | RCT | China | DU (60) | OME (30)  PAN (30) | 26 | 57.0±6.7  57.1±5.6 | *NA* | *NA* | 8 | ① |
| Rehner M  1995 | RCT | Germany | DU (286) | OME (93)  PAN (193) | 187 | 21-84, 47  17-85, 46 | *NA* | *NA* | 4 | ①② |
| Chang FY  1995 | RCT | Taiwan | DU (111) | OME (54)  LAN (57) | 103 | 59.3±12.8  56.4±13.9 | 25  28 | 20  18 | 4 | ①② |
| Ekstrom P  1995 | RCT | Sweden | DU (279) | OME (136)  LAN (143) | 171 | 55.3  54.4 | 61  69 | 57  63 | 4 | ①② |
| Xiao Q  1997 | RCT | China | DU (99) | OME (32)  LAN (41)  FAM (26) | *NA* | 16-68 | *NA* | *NA* | 4 | ①② |
| Xu JY  2001 | RCT | China | DU (150) | OME (75)  RAB (75) | 109 | 40.7±12.1  44.4±10.8 | 47  41 | *NA* | 6 | ①② |
| Hu NZ  2001 | RCT | China | DU (32) | OME (15)  RAB (17) | 26 | 38.0±13.6  41.1±13.2 | 4  5 | *NA* | 4 | ① |
| You QX  2006 | RCT | China | DU (72) | OME (36)  RAB (36) | 54 | 18-75 | *NA* | *NA* | 4 | ①② |
| Xu YH  2006 | RCT | China | DU (68) | OME (34)  RAB (34) | 48 | 34.9  35.1 | *NA* | *NA* | 4 | ①② |
| Dekkers CP  1999 | RCT | Belgium, Germany, Iceland, Ireland, Netherlands, Poland, Spain, Sweden, and United Kingdom | DU (205) | OME (103)  RAB (102) | 134 | 47.8±13.2  47.3±13.5 | 52  47 | 47  48 | 4 | ①② |
| Chen C  2017 | RCT | China | DU (80) | OME (40)  ILA (40) | 48 | 20-60 | *NA* | *NA* | 4 | ①② |
| Liao ZC  2015 | RCT | China | DU (80) | OME (40)  ILA (40) | 44 | 22-76, 61.8 | *NA* | *NA* | 4 | ①② |
| Ho KY  2009 | RCT | Singapore, Malaysia, Australia, Philippines, and Thailand. | DU (203) | OME (104)  ILA (99) | 148 | 51.6±14.4  47.9±13.8 | *NA* | *NA* | 4 | ①② |
| Wang L  2011 | RCT | China | DU (117) | OME (59)  ILA (58) | 83 | 39.2±11.6  39.0±10.8 | *NA* | *NA* | 4 | ①② |
| Wang L  2012 | RCT | China | DU (496) | OME (165)  ILA (331) | 361 | 40.9±11.9  41.2±12.1 | *NA* | *NA* | 4 | ①② |
| Wang XP  2000 | RCT | China | DU (100) | PAN (50)  LAN (50) | 75 | 42±20  43±18 | 8  10 | 4  5 | 4 | ①② |
| Wang P  2013 | RCT | China | DU (220) | LAN (110)  ILA (110) | 117 | 64.6±5.3  65.5±4.5 | *NA* | *NA* | 4 | ①② |
| Li N  2001 | RCT | China | DU (118) | OME (77)  RAN (41) | 78 | 11-66, 36.5 | *NA* | *NA* | 4 | ① |
| Li DF  1994 | RCT | China | DU (39) | OME (21)  RAN (18) | *NA* | 62-81  63-80 | *NA* | *NA* | 4 | ① |
| Bradhan KD  1986 | RCT | England, Italy, and Sweden | DU (69) | OME (34)  RAN (35) | 54 | 18-75, 42.3  21-74, 47.5 | 25  25 | 23  18 | 8 | ①② |
| Chelvam P  1989 | RCT | Malaysia | DU (229) | OME (114)  RAN (115) | 175 | 19-75, 43.9  19-74, 45.1 | 54  42 | 32  29 | 4 | ①② |
| Hui WM  1989 | RCT | Hong Kong | DU (179) | OME (90)  RAN (89) | 132 | 40.2±13.6  38.6±14.2 | 32  24 | 9  7 | 4 | ①② |
| Mulder CJJ  1989 | RCT | Netherlands | DU (181) | OME (91)  RAN (90) | 123 | 49.0±13.4  49.6±16.4 | 60  53 | 66  55 | 8 | ①② |
| McFarland RJ  1990 | RCT | the United Kingdom and Ireland | DU (247) | OME (125)  RAN (122) | *NA* | 46±14  46±15 | 63  71 | *NA* | 4 | ①② |
| Marks IN  1991 | RCT | South Africa | DU (210) | OME (104)  RAN (106) | 145 | 40  40 | 73  79 | 37  32 | 4 | ①② |
| Valenzuela JE  1991 | RCT | the United States | DU (309) | OME (151)  RAN (158) | 225 | 46.3  50.5 | 63  73 | 17  18 | 4 | ①② |
| Lysy J  1992 | RCT | Israel | DU (60) | OME (30)  RAN (30) | 49 | 36.3±2.6  42.3±2.1 | 12  14 | *NA* | 4 | ① |
| Wang CY  1992 | RCT | Taiwan | DU (226) | OME (118)  RAN (108) | 181 | 17-74, 44.5  18-77, 44.5 | 53  63 | 39  38 | 4 | ①② |
| Ahmed W  1993 | RCT | Karachi | DU (41) | OME (21)  RAN (20) | 33 | 38±13  42±15 | 11  6 | *NA* | 4 | ① |
| Zaterka S  1993 | RCT | Brazil | DU (241) | OME (120)  RAN (121) | 150 | 38.1±12.3  37.4±13.1 | 65  61 | 44  45 | 4 | ①② |
| Hallerback B  1998 | RCT | Sweden | DU (393) | OME (195)  RAN (198) | 226 | 49±14  50±15 | 112  110 | 139  129 | 8 | ①② |
| Gu W  2005 | RCT | China | DU (67) | OME (34)  FAM (33) | 46 | 45.1±14.5 46.9±12.1 | *NA* | *NA* | 4 | ①② |
| Tao ZH  1993 | RCT | China | DU (164) | OME (83)  FAM (81) | *NA* | *NA* | *NA* | *NA* | 4 | ① |
| Fave GD  1992 | RCT | Italy | DU (241) | OME (116)  FAM (125) | 172 | 19-76, 47  23-80, 46 | 60  72 | 73  85 | 6 | ①② |
| Kumar TR  1992 | RCT | India | DU (129) | OME (65)  FAM (64) | 105 | 38.5±10.8  38.9±10.0 | *NA* | *NA* | 4 | ①② |
| Misra SC  1993 | RCT | India | DU (60) | OME (30)  FAM (30) | 55 | 40.1±12.5  38.6±9.3 | 16  19 | 9  10 | 4 | ①② |
| Tang SH  2001 | RCT | China | DU (60) | PAN (32)  RAN (28) | *NA* | 18-67, 43.5 | *NA* | *NA* | 4 | ① |
| Judmaier G  1994 | RCT | Austria and Switzerland | DU (202) | PAN (98)  RAN (104) | 138 | 20-66, 46.5  21-70, 44.5 | 33  41 | 5  9 | 4 | ①② |
| Rensburg CJV  1994 | RCT | South Africa | DU (199) | PAN (102)  RAN (97) | 150 | 18-73, 39.5  20-73, 39.0 | *NA* | *NA* | 4 | ①② |
| Cremer M  1995 | RCT | Belgium, France, Italy and the Netherlands | DU (276) | PAN (137)  RAN (139) | 215 | 22-76, 45  18-80, 45 | 63  62 | 22  27 | 4 | ①② |
| Schepp W  1995 | RCT | Germany | DU (266) | PAN (177)  RAN (89) | 183 | 20-85, 45  19-77, 47 | 77  46 | 14  8 | 4 | ①② |
| Meneghelli UG  2000 | RCT | Brazil | DU (222) | PAN (111)  RAN (111) | 121 | 18-70, 36  20-68, 38 | 40  48 | 1  1 | 4 | ①② |
| Londong W  1991 | RCT | Germany | DU (157) | LAN (78)  RAN (79) | 98 | 23-76, 47  18-78, 50 | 43  44 | *NA* | 4 | ①② |
| Hawkey CJ  1993 | RCT | the United Kingdom | DU (193) | LAN (95)  RAN (98) | 139 | 43±14  48±13 | 54  62 | 69  62 | 4 | ①② |
| Lanza F  1994 | RCT | the United States | DU (214) | LAN (84)  RAN (86)  PLA (44) | 123 | ＜40y: 34  ＜40y: 33  ＜40y: 22 | 33  41  22 | 34  49  24 | 4 | ①② |
| Hotz J  1992 | RCT | Germany | DU (264) | LAN (174)  FAM (90) | 171 | 18-76  19-68 | 92  58 | *NA* | 4 | ①② |
| Breiter JR  2000 | RCT | the United States and Canada | DU (376) | RAB (188)  RAN (188) | 249 | 51.5±14.8  49.0±14.7 | 77  72 | 58  61 | 4 | ① |
| David YG  1990 | RCT | the United States | DU (153) | OME (102)  PLA (51) | 117 | 47.5  51 | 53  28 | 11  6 | 4 | ①② |
| Cloud ML  1998 | RCT | the United States | DU (67) | RAB (34)  PLA (33) | *NA* | 50 | 48 | 33 | 4 | ① |
| Alcala-Santaella R  1989 | RCT | Spain | DU (143) | RAN (72)  FAM (71) | 97 | 40.2±13  42.6±14 | 41  43 | 24  27 | 6 | ①② |
| Arthur J Mccullough  1986 | RCT | the United States | DU (514) | RAN (259)  FAM (255) | *NA* | 45.4  43.5 | 57  61 | 47  50 | 8 | ①② |
| Marks IN  1987 | RCT | South Africa | DU (132) | RAN (62)  FAM (70) | 86 | 43.1  43.0 | 40  43 | *NA* | 6 | ①② |
| Dobrilla G  1987 | RCT | Italy | DU (234) | RAN (115)  FAM (119) | 156 | 45.3±11.9  45.8±13.3 | 62  65 | 41  57 | 6 | ① |
| Barbara L  1985 | RCT | Italy | DU (109) | RAN (53)  FAM (56) | 73 | 43.2±10.4  40.2±11.8 | 32  34 | 27  29 | 8 | ①② |
| Simon B  1985 | RCT | Germany | DU (92) | RAN (48)  FAM (44) | 55 | 17-84, 50.0  22-70, 45.6 | 20  22 | *NA* | 8 | ①② |

**n: the number of participants; RCT: randomized controlled trial; DU: Duodenal ulcer; NA: not applicable; OME: omeprazole, 20mg/day; PAN: pantoprazole, 40mg/day; LAN: lansoprazole, 30mg/day; RAB: rabeprazole, 20mg/day; ILA: ilaprazole, 10mg/day; RAN: ranitidine, 300mg/day; FAM: famotidine, 40mg/day; PLA: placebo; y: years; ① 4-week ulcer healing rate; ② adverse events.**

**Table S5**. The results of SUCRA for various strategies from the network meta-analysis

| **SUCRA** | **4-week healing rate** | **Incidence of adverse events** |
| --- | --- | --- |
| OME | 0.5714 | 0.4286 |
| PAN | 0.8571 | 0.2857 |
| LAN | 0.4286 | 0.5714 |
| RAB | 0.5714 | 0.8571 |
| ILA | 1 | 0.8571 |
| RAN | 0.2857 | 0.4286 |
| FAM | 0.1429 | 0.5714 |
| PLA | 0 | 0 |

**SUCRA: the surface under the cumulative ranking; OME: omeprazole, 20mg/day; PAN: pantoprazole, 40mg/day; LAN: lansoprazole, 30mg/day; RAB: rabeprazole, 20mg/day; ILA: ilaprazole, 10mg/day; RAN: ranitidine, 300mg/day; FAM: famotidine, 40mg/day; PLA: placebo**

**Table S6. Odds ratio of various PPIs from the network meta-analysis for 4-week ulcer healing rate (per-protocol data)**.

| or | OME | PAN | LAN | RAB | ILA | RAN | FAM | PLA |
| --- | --- | --- | --- | --- | --- | --- | --- | --- |
| OME | 1.0 | 1.466  (1.042, 2.079) | 0.893  (0.581, 1.395) | 0.912  (0.538, 1.548) | 1.522  (0.944, 1.522) | 0.327  (0.257, 0.414) | 0.254  (0.189, 0.347) | 0.140  (0.080, 0.236) |
| PAN | 0.682  (0.481, 0.960) | 1.0 | 0.615  (0.375, 1.015) | 0.625  (0.341, 1.13) | 1.035  (0.588, 1.829) | 0.224  (0.155, 0.314) | 0.174  (0.115, 0.260) | 0.010  (0.051, 0.174) |
| LAN | 1.12  (0.717, 1.723) | 1.625  (0.986, 2.667) | 1.0 | 1.022  (0.540, 1.869) | 1.692  (0.934, 3.096) | 0.368  (0.232, 0.548) | 0.285  (0.178, 0.440) | 0.156  (0.083, 0.293) |
| RAB | 1.097  (0.646, 1.859) | 1.600  (0.885, 2.933) | 0.979  (0.535, 1.853) | 1.0 | 1.691  (0.827, 3.291) | 0.361  (0.211, 0.597) | 0.278  (0.157, 0.493) | 0.152  (0.081, 0.297) |
| ILA | 0.657  (0.417, 1.06) | 0.967  (0.547, 1.700) | 0.591  (0.323, 1.071) | 0.591  (0.304, 1.209) | 1.0 | 0.213  (0.131, 0.351) | 0.167  (0.096, 0.286) | 0.091  (0.046, 0.186) |
| RAN | 3.058  (2.416, 3.893) | 4.460  (3.182, 6.433) | 2.720  (1.825, 4.308) | 2.771  (1.677, 4.741) | 4.685  (2.846, 7.656) | 1.0 | 0.776  (0.592, 1.021) | 0.424  (0.248, 0.736) |
| FAM | 3.945  (2.885, 5.301) | 5.746  (3.847, 8.690) | 3.513  (2.273, 5.632) | 3.600  (2.031, 6.370) | 5.999  (3.494, 10.370) | 1.289  (0.980, 1.690) | 1.0 | 0.543  (0.309, 0.999) |
| PLA | 7.149  (4.246, 12.470) | 10.460  (5.742, 19.550) | 6.433  (3.420, 12.130) | 6.583  (3.365, 12.430) | 11.000  (5.382, 21.960) | 2.358  (1.359, 4.030) | 1.841  (1.001, 1.841) | 1.0 |

**Each number is an odds ratio (=****row/column), and 95% confidence interval. OME: omeprazole, 20mg/day; PAN: pantoprazole, 40mg/day; LAN: lansoprazole, 30mg/day; RAB: rabeprazole, 20mg/day; ILA: ilaprazole, 10mg/day; RAN: ranitidine, 300mg/day; FAM: famotidine, 40mg/day; PLA: placebo. Green shading: no significant difference; Red shading: significant difference.**

**Table S7. Odds ratio of various PPIs from the network meta-analysis for 4-week ulcer healing rate (trials with lower risk of bias).**

| or | OME | PAN | LAN | RAB | ILA | RAN | FAM | PLA |
| --- | --- | --- | --- | --- | --- | --- | --- | --- |
| OME | 1.0 | 1.340  (0.9134, 1.961) | 0.918  (0.622, 1.370) | 1.077  (0.594, 1.980) | 1.193  (0.720, 1.976) | 0.461  (0.365, 0.574) | 0.380  (0.277, 0.523) | 0.160  (0.090, 0.283) |
| PAN | 0.747  (0.510, 1.095) | 1.0 | 0.686  (0.415, 1.132) | 0.806  (0.421, 1.572) | 0.890  (0.473, 1.668) | 0.342  (0.245, 0.475) | 0.284  (0.185, 0.431) | 0.119  (0.062, 0.235) |
| LAN | 1.089  (0.730, 1.608) | 1.459  (0.883, 2.412) | 1.0 | 1.166  (0.619, 2.310) | 1.294  (0.683, 2.498) | 0.498  (0.343, 0.726) | 0.411  (0.271, 0.632) | 0.174  (0.094, 0.321) |
| RAB | 0.928  (0.505, 1.684) | 1.241  (0.731, 2.378) | 0.857  (0.433, 1.615) | 1.0 | 1.104  (0.491, 2.453) | 0.428  (0.236, 0.753) | 0.354  (0.1864, 0.6633) | 0.1479  (0.0655, 0.3252) |
| ILA | 0.8382  (0.5061, 1.389) | 1.124  (0.5995, 2.114) | 0.7731  (0.4004, 1.465) | 0.9057  (0.4077, 2.038) | 1.0 | 0.3848  (0.2203, 0.6697) | 0.320  (0.175, 0.574) | 0.135  (0.062, 0.283) |
| RAN | 2.171  (1.742, 2.742) | 2.924  (2.104, 4.077) | 2.010  (1.378, 2.920) | 2.336  (1.328, 2.336) | 2.599  (1.493, 4.540) | 1.0 | 0.828  (0.632, 1.091) | 0.345  (0.198, 0.621) |
| FAM | 2.633  (1.913, 3.608) | 3.516  (2.320, 5.399) | 2.432  (1.584, 3.692) | 2.827  (1.508, 5.366) | 3.123  (1.741, 5.709) | 1.208  (0.917, 1.582) | 1.0 | 0.417  (0.227, 0.780) |
| PLA | 6.272  (3.532, 11.100) | 8.401  (4.263, 16.240) | 5.744  (3.114, 10.600) | 6.761  (3.076, 15.280) | 7.414  (3.539, 16.060) | 2.899  (1.610, 5.064) | 2.401  (1.283, 4.408) | 1.0 |

**Each number is an odds ratio (=row/column), and 95%** **confidence interval. OME: omeprazole, 20mg/day; PAN: pantoprazole, 40mg/day; LAN: lansoprazole, 30mg/day; RAB: rabeprazole, 20mg/day; ILA: ilaprazole, 10mg/day; RAN: ranitidine, 300mg/day; FAM: famotidine, 40mg/day; PLA: placebo. Green shading: no significant difference; Red shading: significant difference.**

**Table S8. Odds ratio of various PPIs from the network meta-analysis for adverse events ((trials with lower risk of bias)).**

| or | OME | PAN | LAN | RAB | ILA | RAN | FAM | PLA |
| --- | --- | --- | --- | --- | --- | --- | --- | --- |
| OME | 1.0 | 1.224  (0.721, 2.123) | 1.009  (0.627, 1.696) | 0.848  (0.388, 1.781) | 0.855  (0.497, 1.465) | 1.028  (0.735, 1.434) | 0.898  (0.504, 1.586) | 1.708  (0.708, 3.869) |
| PAN | 0.817  (0.471, 1.387) | 1.0 | 0.827  (0.424, 1.664) | 0.688  (0.263, 1.719) | 0.698  (0.328, 1.484) | 0.839  (0.509, 1.368) | 0.735  (0.368, 1.451) | 1.390  (0.506, 3.626) |
| LAN | 0.991  (0.590, 1.595) | 1.209  (0.601, 2.357) | 1.0 | 0.834  (0.322, 2.043) | 0.851  (0.397, 1.744) | 1.017  (0.601, 1.637) | 0.891  (0.467, 1.630) | 1.689  (0.642, 4.240) |
| RAB | 1.179  (0.562, 2.577) | 1.453  (0.582, 3.799) | 1.199  (0.489, 3.105) | 1.0 | 1.013  (0.404, 2.599) | 1.216  (0.536, 2.822) | 1.072  (0.416, 2.830) | 2.038  (0.635, 6.165) |
| ILA | 1.169  (0.683, 2.006) | 1.433  (0.674, 3.054) | 1.175  (0.574, 2.521) | 0.988  (0.385, 2.476) | 1.0 | 1.199  (0.635, 2.272) | 1.050  (0.488, 2.273) | 1.990  (0.710, 5.302) |
| RAN | 0.973  (0.698, 1.361) | 1.192  (0.731, 1.192) | 0.983  (0.611, 1.665) | 0.823  (0.354, 1.866) | 0.834  (0.440, 1.575) | 1.0 | 0.875  (0.530, 1.490) | 1.663  (0.669, 3.592) |
| FAM | 1.114  (0.631, 1.983) | 1.360  (0.689, 2.717) | 1.123  (0.614, 2.140) | 0.933  (0.354, 2.404) | 0.953  (0.440, 2.049) | 1.143  (0.671, 1.889) | 1.0 | 1.894  (0.693, 5.065) |
| PLA | 0.585  (0.259, 1.412) | 0.720  (0.276, 1.976) | 0.592  (0.236, 1.557) | 0.491  (0.162, 1.574) | 0.503  (0.189, 1.409) | 0.601  (0.253, 1.495) | 0.528  (0.197, 1.442) | 1.0 |

**Each number is an odds ratio (=row/column), and 95% confidence interval. OME: omeprazole, 20mg/day; PAN: pantoprazole, 40mg/day; LAN: lansoprazole, 30mg/day; RAB: rabeprazole, 20mg/day; ILA: ilaprazole, 10mg/day; RAN: ranitidine, 300mg/day; FAM: famotidine, 40mg/day; PLA: placebo. Green shading: no significant difference.**

**Table S9. Odds ratio of various PPIs from the network meta-analysis for 4-week ulcer healing rate in Chinese**.

| or | OME | PAN | LAN | RAB | ILA | RAN | FAM |
| --- | --- | --- | --- | --- | --- | --- | --- |
| OME | 1.0 | 1.252  (0.782, 2.025) | 1.248  (0.627, 2.506) | 1.264  (0.364, 4.821) | 1.815  (1.122, 3.093) | 0.365  (0.215, 0.600) | 0.169  (0.069, 0.364) |
| PAN | 0.799  (0.494, 1.278) | 1.0 | 0.992  (0.460, 2.089) | 0.999  (0.264, 4.071) | 1.452  (0.740, 2.887) | 0.291  (0.148, 0.541) | 0.133  (0.050, 0.323) |
| LAN | 0.801  (0.399, 1.596) | 1.008  (0.479, 2.175) | 1.0 | 1.010  (0.246, 4.606) | 1.469  (0.672, 3.204) | 0.294  (0.126, 0.684) | 0.135  (0.049, 0.346) |
| RAB | 0.791  (0.207, 2.749) | 1.001  (0.246, 3.788) | 0.990  (0.217, 4.061) | 1.0 | 1.453  (0.349, 5.691) | 0.288  (0.069, 1.105) | 0.134  (0.027, 0.562) |
| ILA | 0.551  (0.323, 0.892) | 0.689  (0.346, 1.352) | 0.681  (0.312, 1.488) | 0.688  (0.176, 2.864) | 1.0 | 0.198  (0.094, 0.400) | 0.092  (0.033, 0.233) |
| RAN | 2.740  (1.666, 4.650) | 3.441  (1.850, 6.754) | 3.401  (1.461, 7.970) | 3.471  (0.905, 14.440) | 5.044  (2.502, 10.590) | 1.0 | 0.467  (0.162, 1.167) |
| FAM | 5.918  (2.751, 14.410) | 7.528  (3.097, 20.070) | 7.421  (2.889, 20.520) | 7.479  (1.781, 37.450) | 10.900  (4.292, 30.190) | 2.141  (0.857, 6.170) | 1.0 |

**Each number is an odds ratio (=row/column), and 95% confidence interval. OME: omeprazole, 20mg/day; PAN: pantoprazole, 40mg/day; LAN: lansoprazole, 30mg/day; RAB: rabeprazole, 20mg/day; ILA: ilaprazole, 10mg/day; RAN: ranitidine, 300mg/day; FAM: famotidine, 40mg/day. Green shading: no significant difference; Red shading: significant difference.**

**Table S10. Odds ratio of various PPIs from the network meta-analysis for 4-week ulcer healing rate in non-Chinese**.

| or | OME | PAN | LAN | RAB | ILA | RAN | FAM | PLA |
| --- | --- | --- | --- | --- | --- | --- | --- | --- |
| OME | 1.0 | 1.301  (0.891, 1.925) | 0.850  (0.552, 1.278) | 1.015  (0.589, 1.806) | 0.970  (0.437, 2.135) | 0.446  (0.349, 0.560) | 0.372  (0.268, 0.508) | 0.156  (0.092, 0.267) |
| PAN | 0.769  (0.520, 1.122) | 1.0 | 0.653  (0.389, 1.074) | 0.779  (0.428, 1.480) | 0.744  (0.309, 1.787) | 0.343  (0.244, 0.474) | 0.285  (0.185, 0.433) | 0.120  (0.064, 0.228) |
| LAN | 1.176  (0.783, 1.811) | 1.530  (0.479, 2.574) | 1.0 | 1.195  (0.648, 2.352) | 1.145  (0.482, 2.802) | 0.525  (0.354, 0.778) | 0.441  (0.281, 0.680) | 0.185  (0.100, 0.337) |
| RAB | 0.985  (0.554, 1.698) | 1.284  (0.676, 2.338) | 0.837  (0.425, 1.544) | 1.0 | 0.952  (0.360, 2.446) | 0.439  (0.250, 0.728) | 0.364  (0.199, 0.645) | 0.154  (0.077, 0.301) |
| ILA | 1.031  (0.468, 2.287) | 1.344  (0.560, 3.236) | 0.874  (0.357, 2.075) | 1.051  (0.409, 2.778) | 1.0 | 0.458  (0.201, 1.066) | 0.383  (0.163, 0.896) | 0.162  (0.063, 0.410) |
| RAN | 2.244  (1.786, 2.863) | 2.917  (2.112, 4.102) | 1.906  (1.285, 2.824) | 2.279  (1.374, 4.006) | 2.182  (0.938, 4.964) | 1.0 | 0.834  (0.641, 1.091) | 0.350  (0.206, 0.602) |
| FAM | 2.689  (1.969, 3.726) | 3.506  (2.311, 5.401) | 2.270  (1.471, 3.562) | 2.748  (1.552, 5.020) | 2.614  (1.116, 6.143) | 0.917  (0.003, 1.199) | 1.0 | 0.421  (0.234, 0.750) |
| PLA | 6.410  (3.746, 10.900) | 8.356  (4.382, 15.650) | 5.415  (2.967, 10.010) | 6.500  (3.323, 13.070) | 6.191  (2.439, 16.000) | 2.857  (1.661, 4.863) | 2.374  (1.334, 4.270) | 1.0 |

**Each number is an odds ratio (=row/column), and 95% confidence interval. OME: omeprazole, 20mg/day; PAN: pantoprazole, 40mg/day; LAN: lansoprazole, 30mg/day; RAB: rabeprazole, 20mg/day; ILA: ilaprazole, 10mg/day; RAN: ranitidine, 300mg/day; FAM: famotidine, 40mg/day; PLA: placebo. Green shading: no significant difference; Red shading: significant difference.**

**Table S11. Odds ratio of various PPIs from the network meta-analysis for adverse events in Chinese.**

| or | OME | PAN | LAN | RAB | ILA | RAN | FAM |
| --- | --- | --- | --- | --- | --- | --- | --- |
| OME | 1.0 | 0.791  (0.340, 1.695) | 0.718  (0.286, 1.669) | 0.470  (0.105, 1.756) | 0..547  (0.239, 1.080) | 1.147  (0.295, 4.232) | 0.954  (0.298, 2.947) |
| PAN | 1.264  (0.590, 2.946) | 1.0 | 0.915  (0.330, 2.580) | 0.590  (0.112, 2.853) | 0.690  (0.237, 1.987) | 1.461  (0.315, 6.767) | 1.217  (0.329, 4.756) |
| LAN | 1.393  (0.599, 3.495) | 1.093  (0.388, 3.034) | 1.0 | 0.652  (0.116, 3.122) | 0.761  (0.262, 2.093) | 1.590  (0.326, 7.625) | 1.321  (0.393, 4.648) |
| RAB | 2.128  (0570, 9.577) | 1.697  (0.351, 8.975) | 1.533  (0.320, 8.647) | 1.0 | 1.169  (0.250, 5.911) | 2.478  (0.371, 17.320) | 2.049  (0.356, 13.370) |
| ILA | 1.827  (0.926, 4.190) | 1.450  (0.504, 4.219) | 1.313  (0.478, 3.818) | 0.856  (0.169, 4.008) | 1.0 | 2.111  (0.468, 9.446) | 1.752  (0.471, 6.980) |
| RAN | 0.872  (0.236, 3.395) | 0.685  (0.148, 3.172) | 0.629  (0.131, 3.065) | 0.404  (0.058, 2.698) | 0.474  (0.106, 2.138) | 1.0 | 0.837  (0.151, 4.790) |
| FAM | 1.048  (0.339, 3.356) | 0.822  (0.210, 3.041) | 0.757  (0.215, 2.543) | 0.488  (0.075, 2.813) | 0.571  (0.143, 2.125) | 1.195  (0.209, 6.639) | 1.0 |

**Each number is an odds ratio (=row/column), and 95% confidence interval. OME: omeprazole, 20mg/day; PAN: pantoprazole, 40mg/day; LAN: lansoprazole, 30mg/day; RAB: rabeprazole, 20mg/day; ILA: ilaprazole, 10mg/day; RAN: ranitidine, 300mg/day; FAM: famotidine, 40mg/day. Green shading: no significant difference.**

**Table S12. Odds ratio of various PPIs from the network meta-analysis for adverse events in non-Chinese.**

| or | OME | PAN | LAN | RAB | ILA | RAN | FAM | PLA |
| --- | --- | --- | --- | --- | --- | --- | --- | --- |
| OME | 1.0 | 1.193  (0.704, 2.041) | 0.955  (0.584, 1.684) | 0.965  (0.392, 2.271) | 1.052  (0.450, 2.588) | 0.980  (0.717, 1.349) | 0.864  (0.500, 1.536) | 1.646  (0.659, 3.902) |
| PAN | 0.838  (0.490, 1.420) | 1.0 | 0.805  (0.401, 1.644) | 0.811  (0.281, 2.186) | 0.888  (0.321, 2.511) | 0.825  (0.509, 1.331) | 0.732  (0.365, 1.482) | 1.379  (0.481, 3.749) |
| LAN | 1.048  (0.594, 1.712) | 1.243  (0.608, 2.495) | 1.0 | 1.001  (0.342, 2.593) | 1.108  (0.386, 3.023) | 1.032  (0.596, 1.642) | 0.912  (0.473, 1.665) | 1.706  (0.626, 4.168) |
| RAB | 1.036  (0.441, 2.550) | 1.234  (0.458, 3.563) | 0.999  (0.386, 2.928) | 1.0 | 1.093  (0.325, 3.968) | 1.021  (0.412, 2.677) | 0.896  (0.332, 2.677) | 1.709  (0.501, 5.774) |
| ILA | 0.951  (0.386, 2.225) | 1.127  (0.398, 3.114) | 0.903  (0.331, 2.594) | 0.915  (0.252, 3.082) | 1.0 | 0.933  (0.361, 2.308) | 0.818  (0.287, 2.307) | 1.551  (0.433, 5.115) |
| RAN | 1.021  (0.741, 1.396) | 1.212  (0.751, 1.965) | 0.969  (0.609, 1.679) | 0.979  (0.374, 2.427) | 1.072  (0.433, 2.771) | 1.0 | 0.882  (0.538, 1.501) | 1.672  (0.650, 4.020) |
| FAM | 1.158  (0.651, 2.001) | 1.366  (0.675, 2.742) | 1.097  (0.601, 2.113) | 1.117  (0.374, 3.013) | 1.223  (0.434, 3.489) | 1.134  (0.666, 1.859) | 1.0 | 1.904  (0.667, 5.236) |
| PLA | 0.608  (0.256, 1.519) | 0.725  (0.267, 2.081) | 0.586  (0.240, 1.599) | 0.585  (0.173, 1.998) | 0.645  (0.196, 2.310) | 0.598  (0.249, 1.538) | 0.525  (0.191, 1.499) | 1.0 |

**Each number is an odds ratio (=row/column), and 95% confidence interval. OME: omeprazole, 20mg/day; PAN: pantoprazole, 40mg/day; LAN: lansoprazole, 30mg/day; RAB: rabeprazole, 20mg/day; ILA: ilaprazole, 10mg/day; RAN: ranitidine, 300mg/day; FAM: famotidine, 40mg/day; PLA: placebo. Green shading: no significant difference.**

**Table S13. Base-case value for the decision model and ranges used in cost-effectiveness sensitivity analyses**

| Parameter | Base case | Sensitivity Range | References |
| --- | --- | --- | --- |
| **Model inputs** |  |  |  |
| **OR of DU healing for other PPIs versus omeprazole** |  |  |  |
| Pantoprazole | 1.252 | 0.782-2.025 (Lognormal) | NMA. Table S9. |
| Lansoprazole | 1.248 | 0.627-2.506 (Lognormal) | NMA. Table S9. |
| Rabeprazole | 1.264 | 0.364-4.821 (Lognormal) | NMA. Table S9. |
| Ilaprazole | 1.815 | 1.122-3.093 (Lognormal) | NMA. Table S9. |
|  |  |  |  |
| **Probability of DU healing** |  |  |  |
| Omeprazole | 0.9249 | 0.9040-0.9463 (Beta) | Single-arm meta-analysis. Figure S7 |
| Pantoprazole | 0.9391 | 0.8804-0.9727 (Beta) | Transformed from NMA |
| Lansoprazole | 0.9389 | 0.8552-0.9779 (Beta) | Transformed from NMA |
| Rabeprazole | 0.9396 | 0.7741-0.9884 (Beta) | Transformed from NMA |
| Ilaprazole | 0.9572 | 0.9135-0.9820 (Beta) | Transformed from NMA |
|  |  |  |  |
| **Probability of AEs** |  |  |  |
| Headache for Omeprazole | 0.0257 |  | Table 3 |
| Headache for Pantoprazole | 0.0179 |  | Table 3 |
| Headache for Lansoprazole | 0.0188 |  | Table 3 |
| Headache for Rabeprazole | 0.0349 |  | Table 3 |
| Headache for Ilaprazole | 0.0046 |  | Table 3 |
| Diarrhea for Omeprazole | 0.0158 |  | Table 3 |
| Diarrhea for Pantoprazole | 0.0154 |  | Table 3 |
| Diarrhea for Lansoprazole | 0.0116 |  | Table 3 |
| Diarrhea for Rabeprazole | 0.0230 |  | Table 3 |
| Diarrhea for Ilaprazole | 0.0183 |  | Table 3 |
| Abnormal liver function for Omeprazole | 0.0123 |  | Table 3 |
| Abnormal liver function for Pantoprazole | 0.0102 |  | Table 3 |
| Abnormal liver function for Lansoprazole | 0.0100 |  | Assumed |
| Abnormal liver function for Rabeprazole | 0.0100 |  | Assumed |
| Abnormal liver function for Ilaprazole | 0.0363 |  | Table 3 |
|  |  |  |  |
| **Cost** |  |  |  |
| Omeprazole (US$/20mg daily po) | 0.1519 | 0.0204-2.4943 (Normal)* | NHFPC of China |
| Pantoprazole (US$/40mg daily po) | 0.4215 | 0.1746-1.6023 (Normal)* | NHFPC of China |
| Lansoprazole (US$/30mg daily po) | 1.0583 | 0.5494-3.4283 (Normal)* | NHFPC of China |
| Rabeprazole (US$/20mg daily po) | 1.4945 | 1.1481-1.7823 (Gamma) | NHFPC of China |
| Ilaprazole (US$/10mg daily po) | 5.3198 | 5.2111-5.4286 (Gamma) | NHFPC of China |
|  |  |  |  |
| **Management of adverse events** |  |  |  |
| Headache (US$) | 15.87 |  | Reference |
| Diarrhea (US$) | 15.87 |  | Reference |
| Abnormal liver function (US$) | 31.75 |  | Reference |
|  |  |  |  |
| **Other parameter** |  |  |  |
| Baseline health state utility value of general population of China |  |  |  |
| Male | 0.834 |  | Reference |
| Female | 0.815 |  | Reference |
| Disutility of DU | -0.11 |  | Reference |

**DU: Duodenal ulcer; NMA: network meta-analysis; NHFPC: National Health and Family Planning Commission of the People’s Republic; *: the distribution of data was log_0.5_ transformed.**


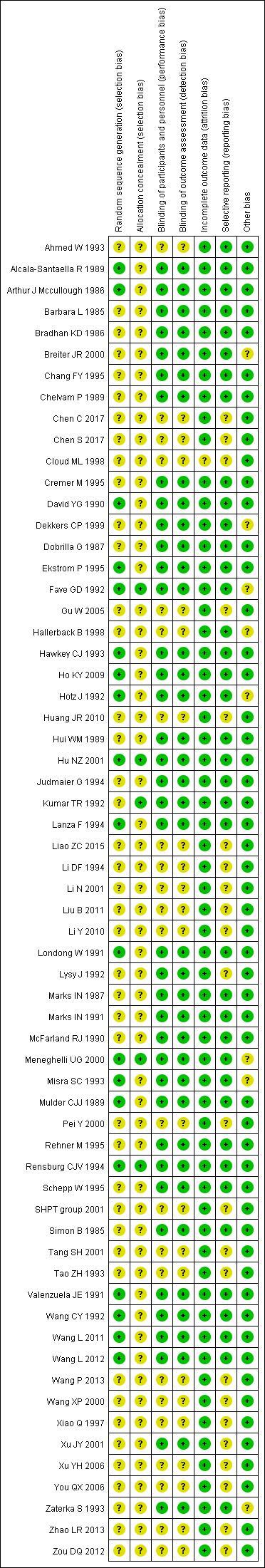


**Figure S1. Risk of bias summary**


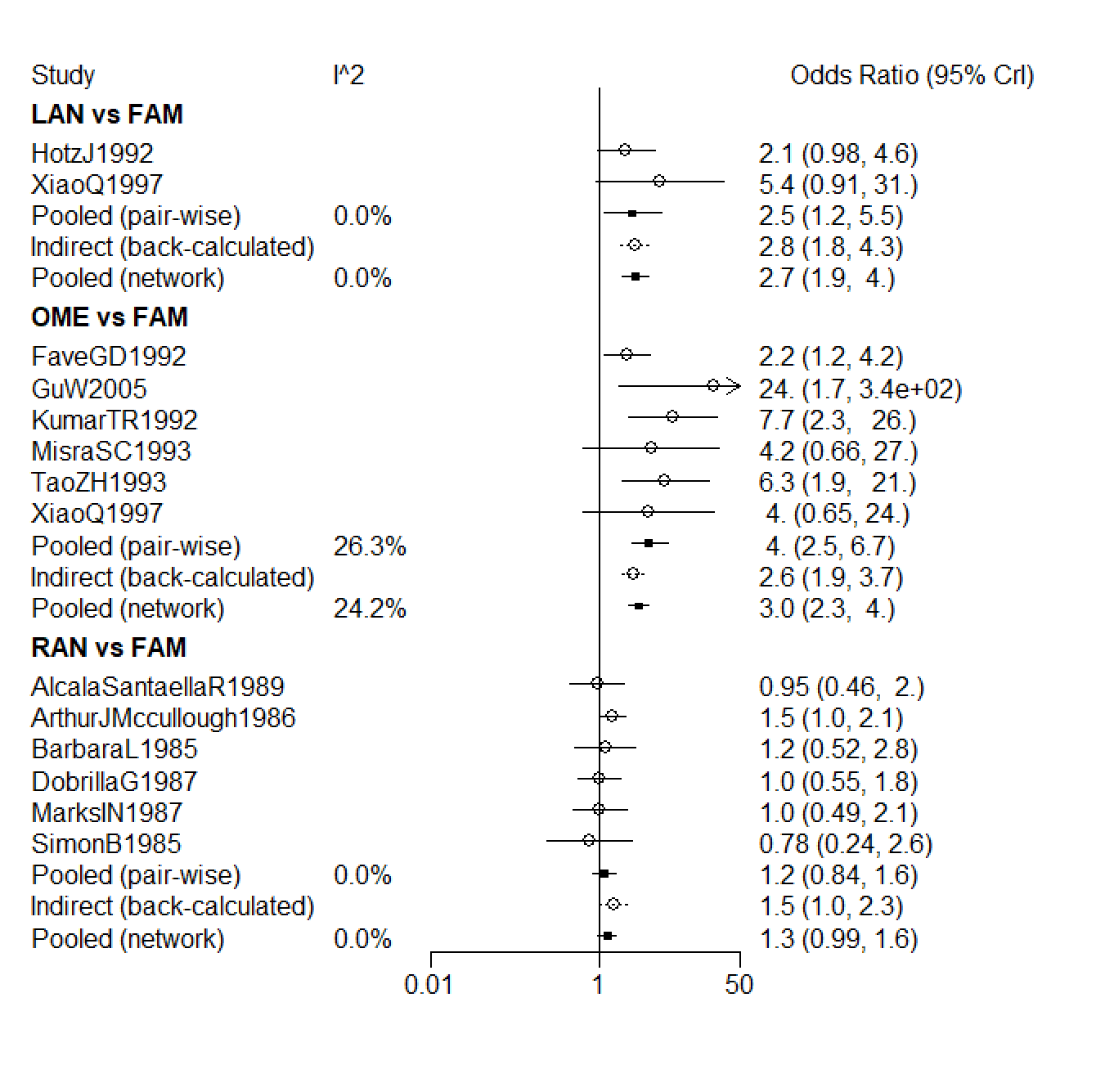


**Figure S2(a). Forest plot of network meta-analysis results for 4-week ulcer healing rate.**


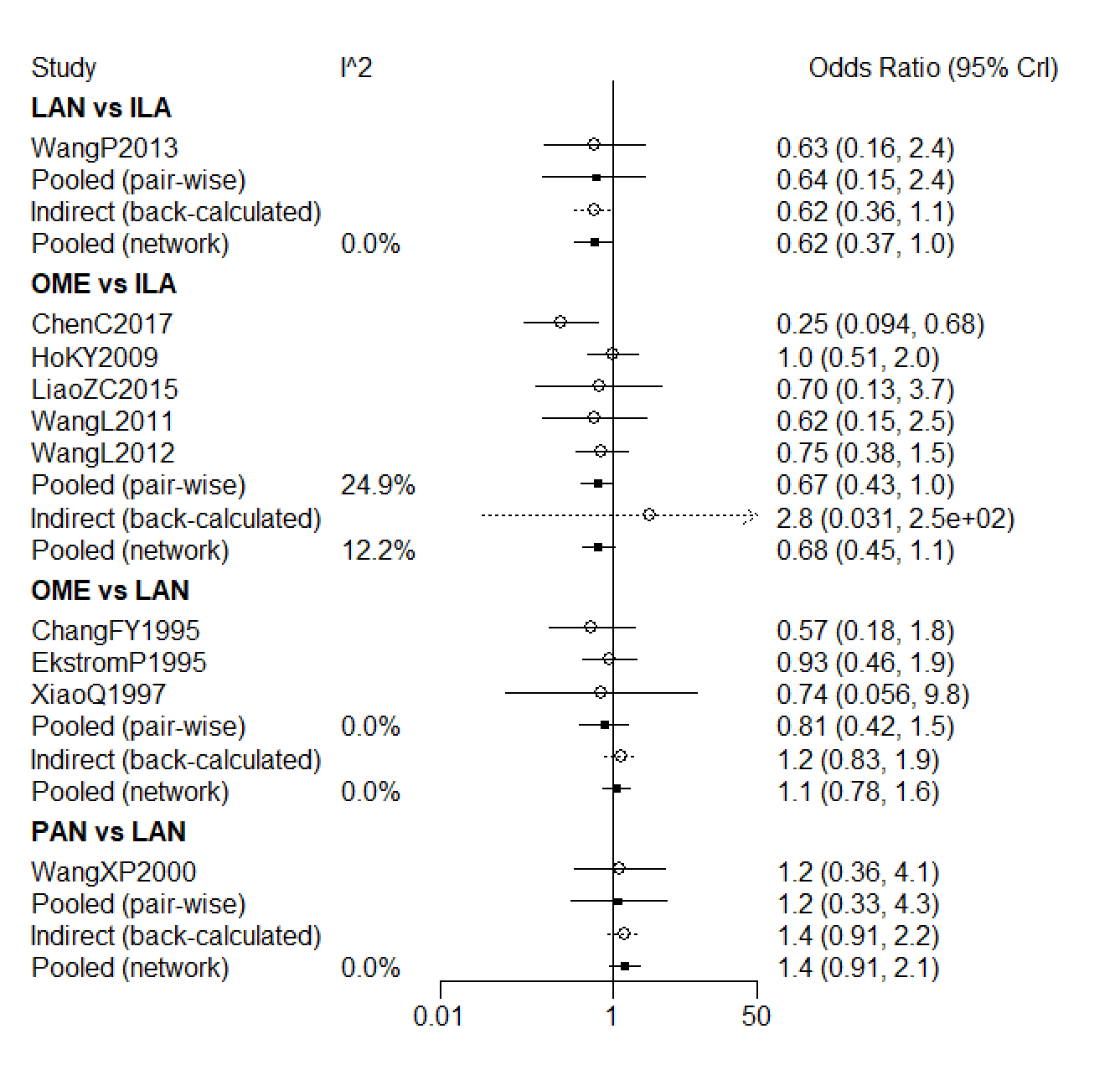


**Figure S2(b). Forest plot of network meta-analysis results for 4-week ulcer healing rate.**


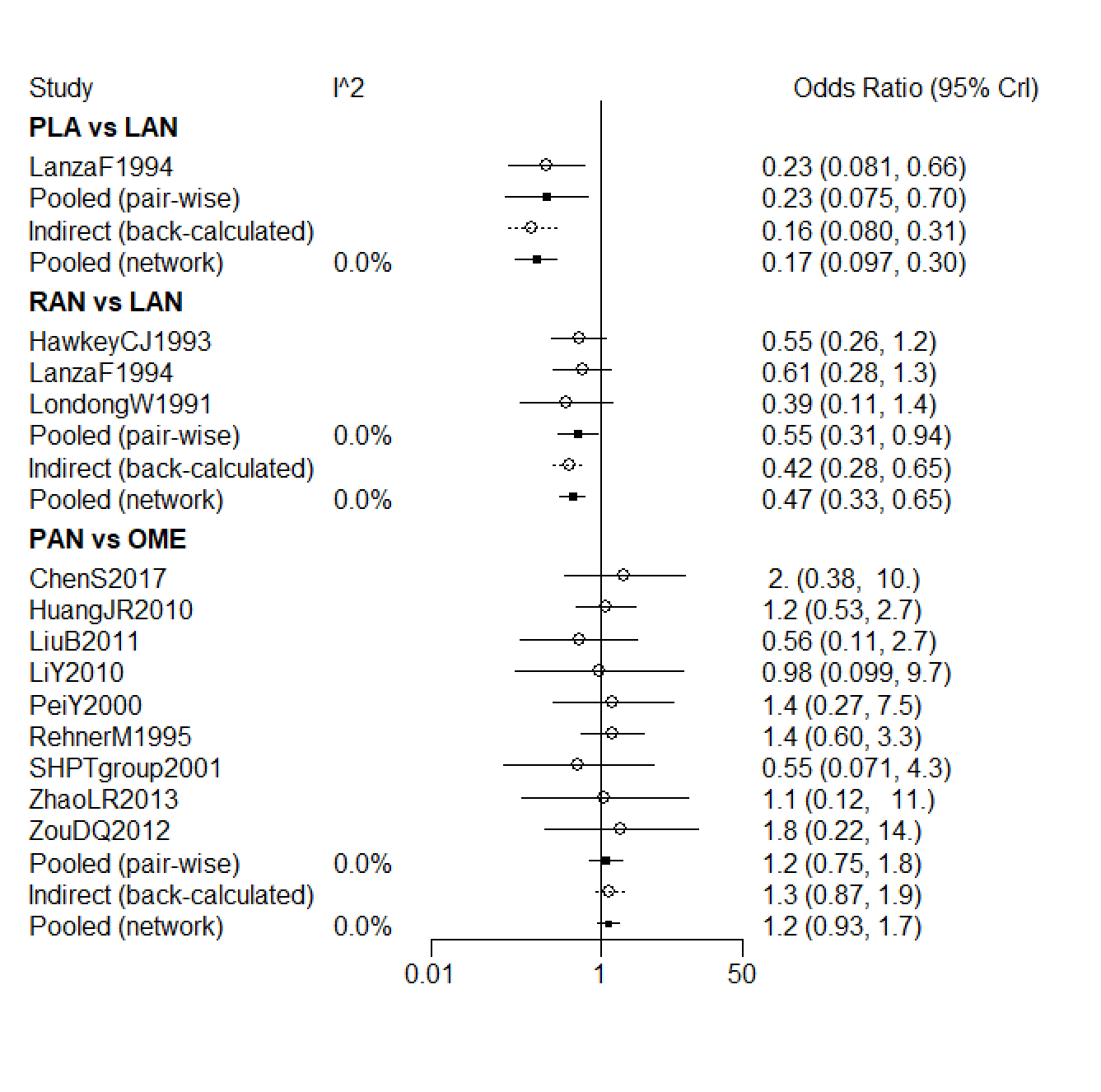


**Figure S2(c). Forest plot of network meta-analysis results for 4-week ulcer healing rate.**


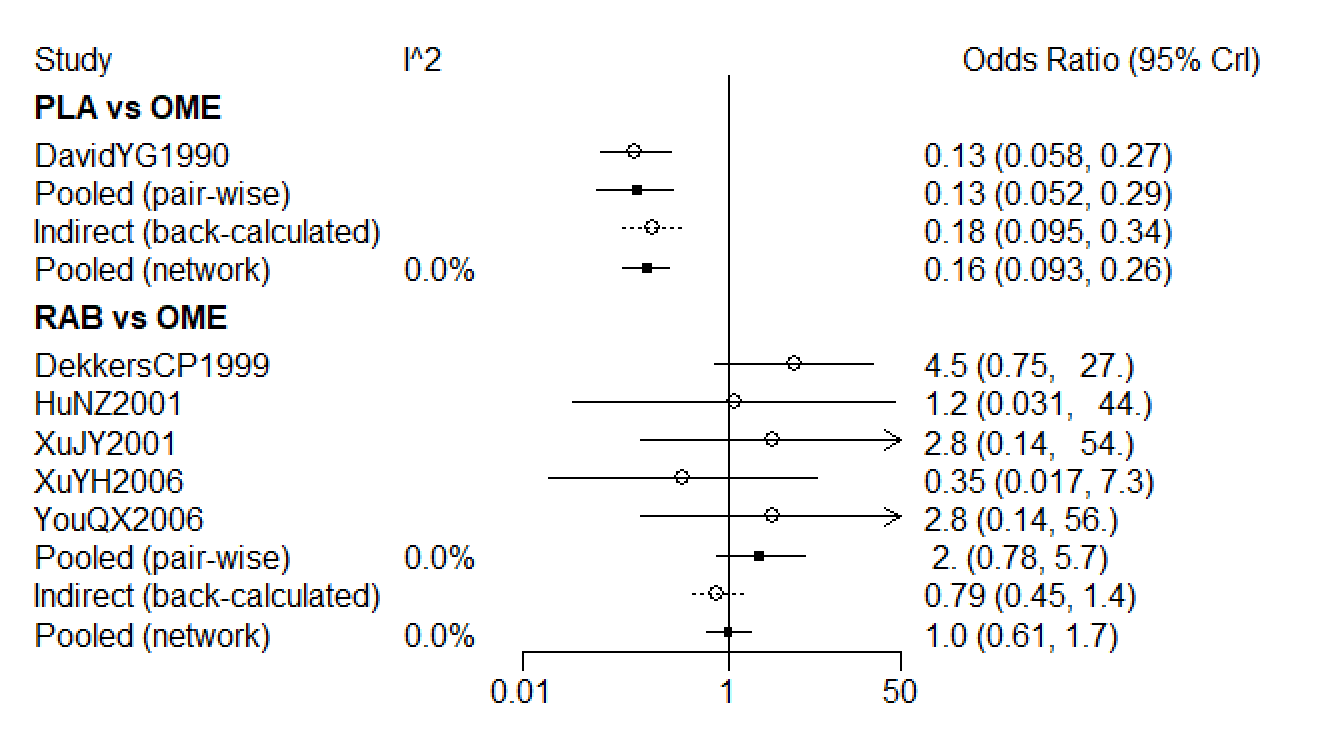


**Figure S2(d). Forest plot of network meta-analysis results for 4-week ulcer healing rate.**


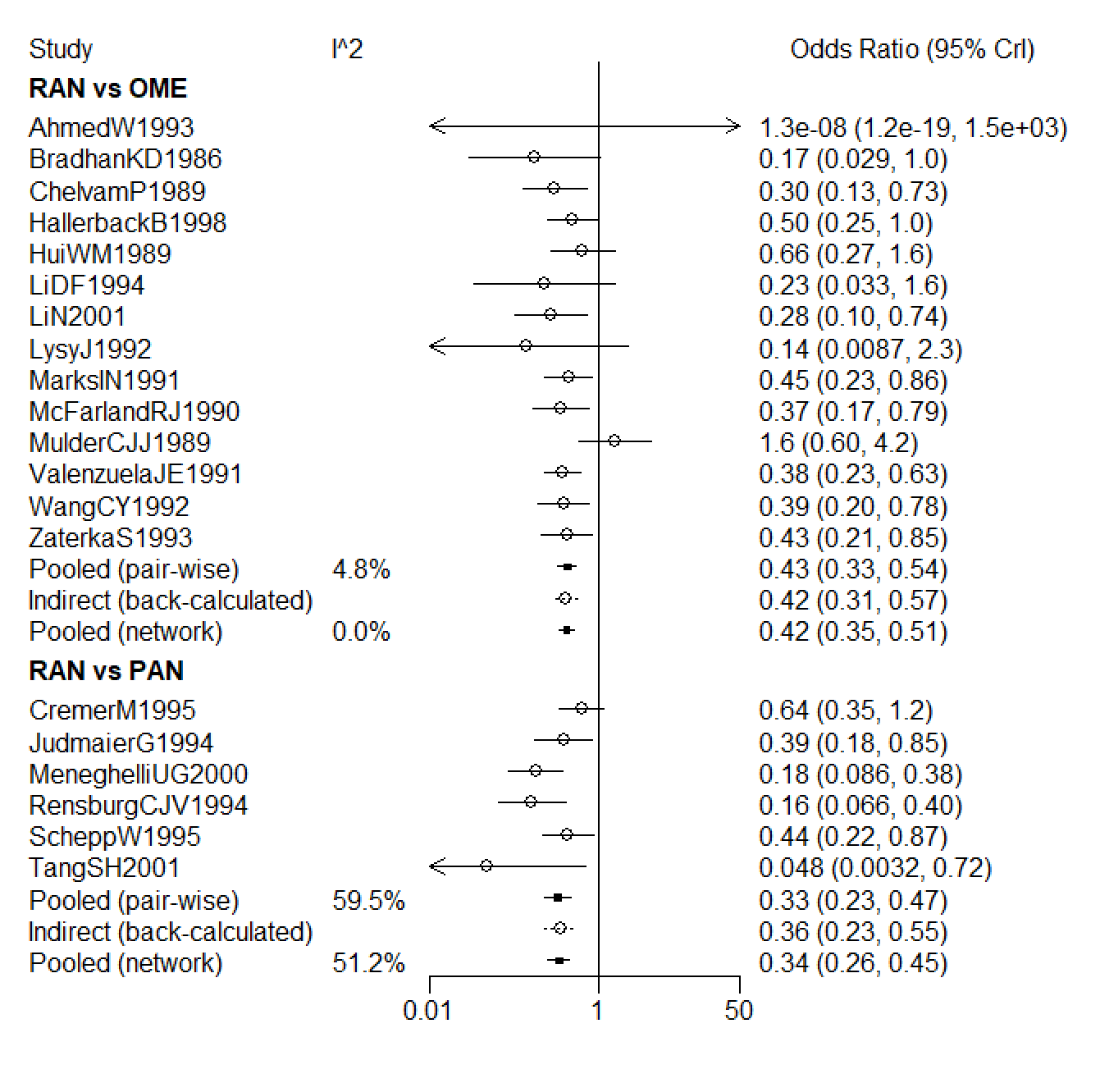


**Figure S2(f). Forest plot of network meta-analysis results for 4-week ulcer healing rate.**


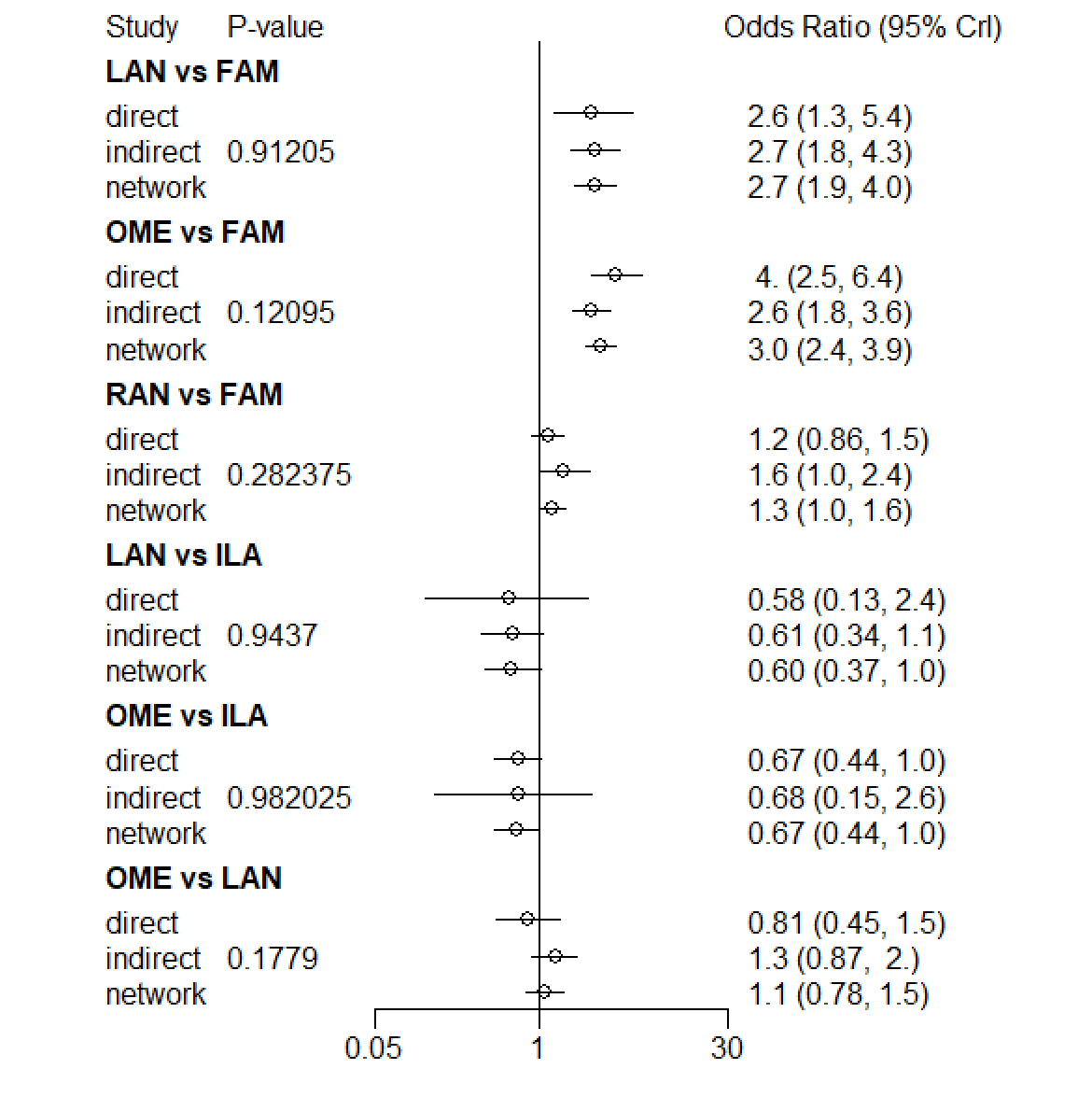


**Figure S3(a). Assessment of network consistency for 4-week ulcer healing rate. Only comparisons for which both pairwise and indirect estimates are available.**


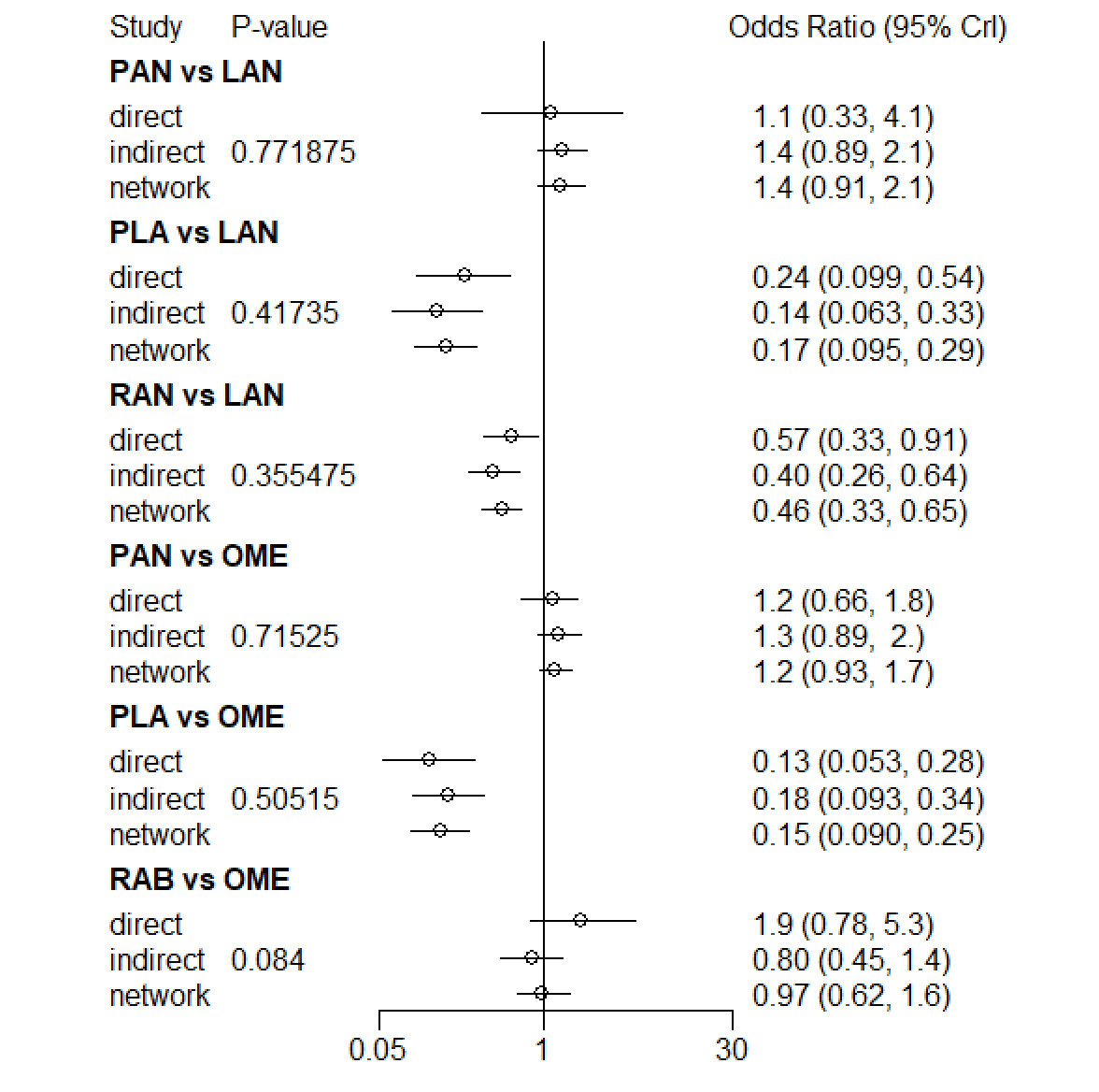


**Figure S3(b). Assessment of network consistency for 4-week ulcer healing rate. Only comparisons for which both pairwise and indirect estimates are available.**


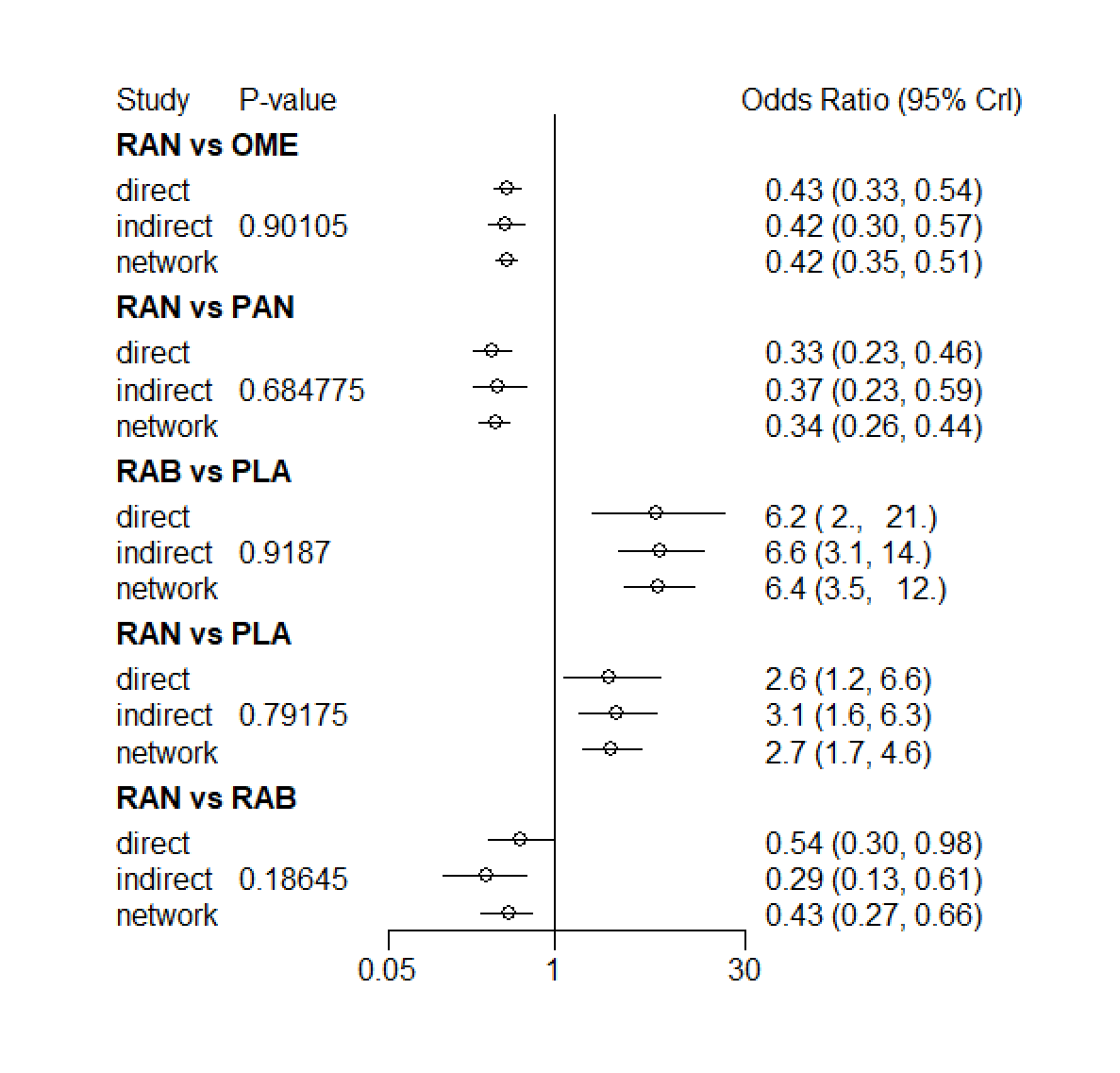


**Figure S3(c). Assessment of network consistency for 4-week ulcer healing rate. Only comparisons for which both pairwise and indirect estimates are available.**


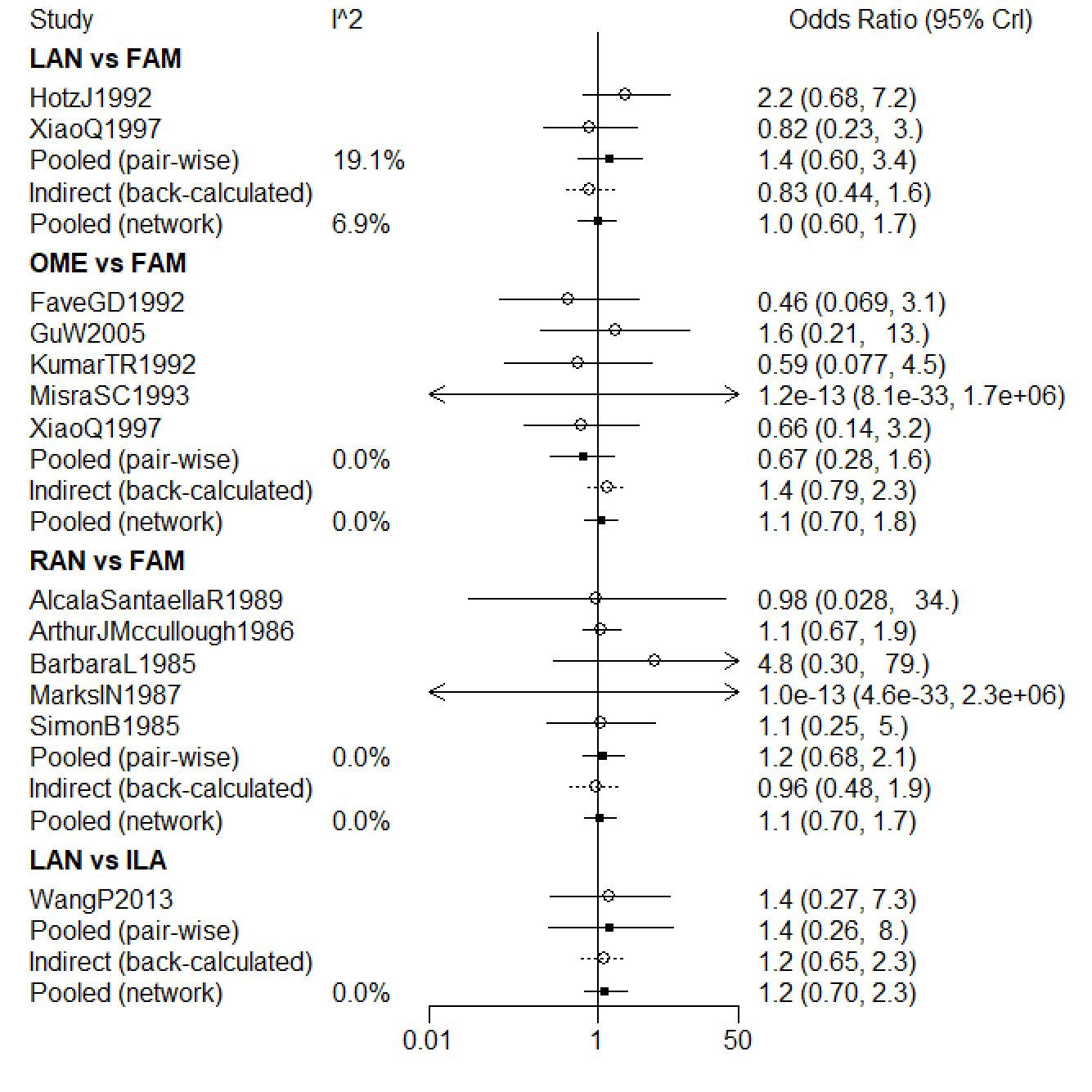


**Figure S4(a). Forest plot of network meta-analysis results for adverse events.**


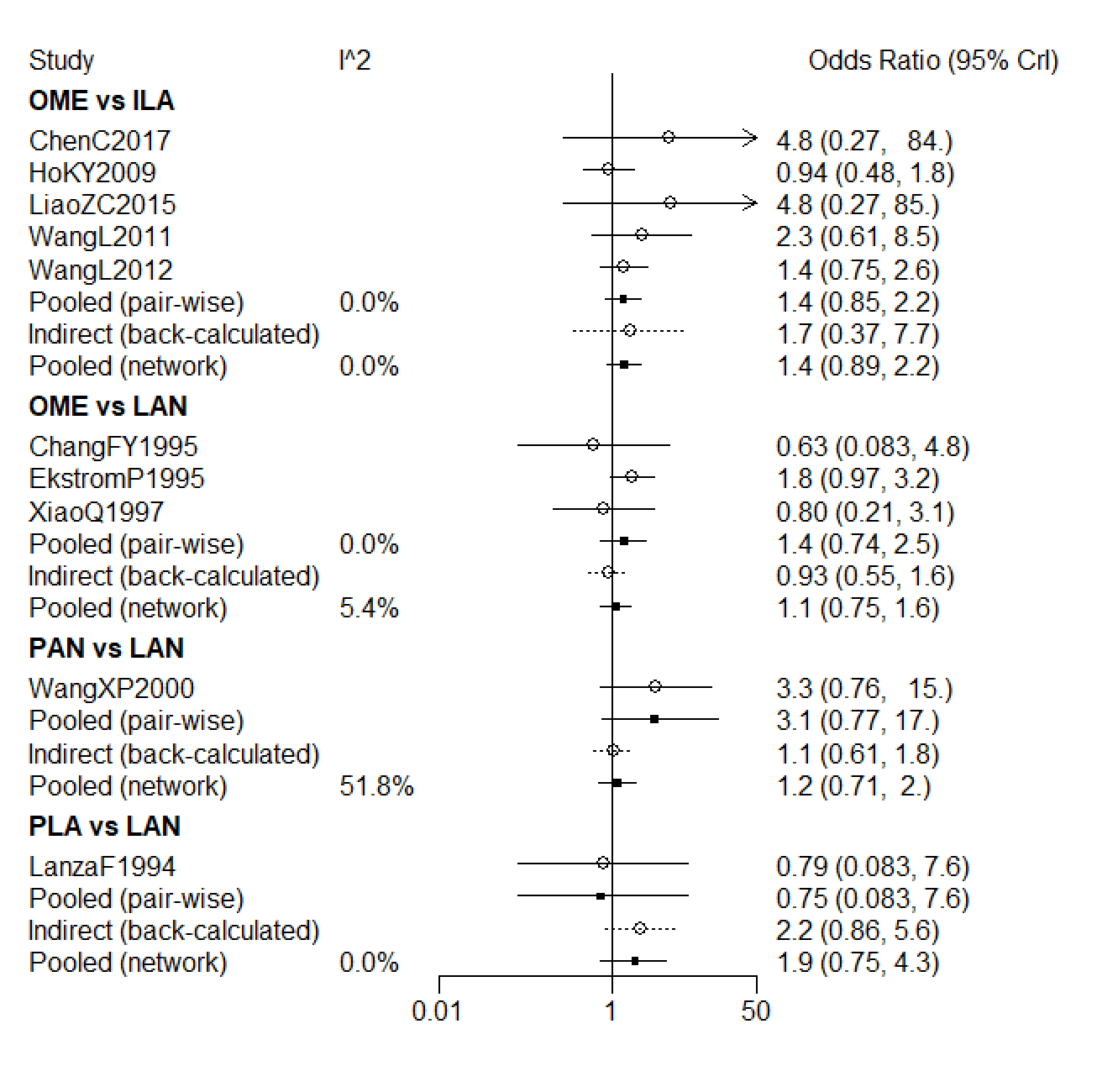


**Figure S4(b). Forest plot of network meta-analysis results for adverse events.**


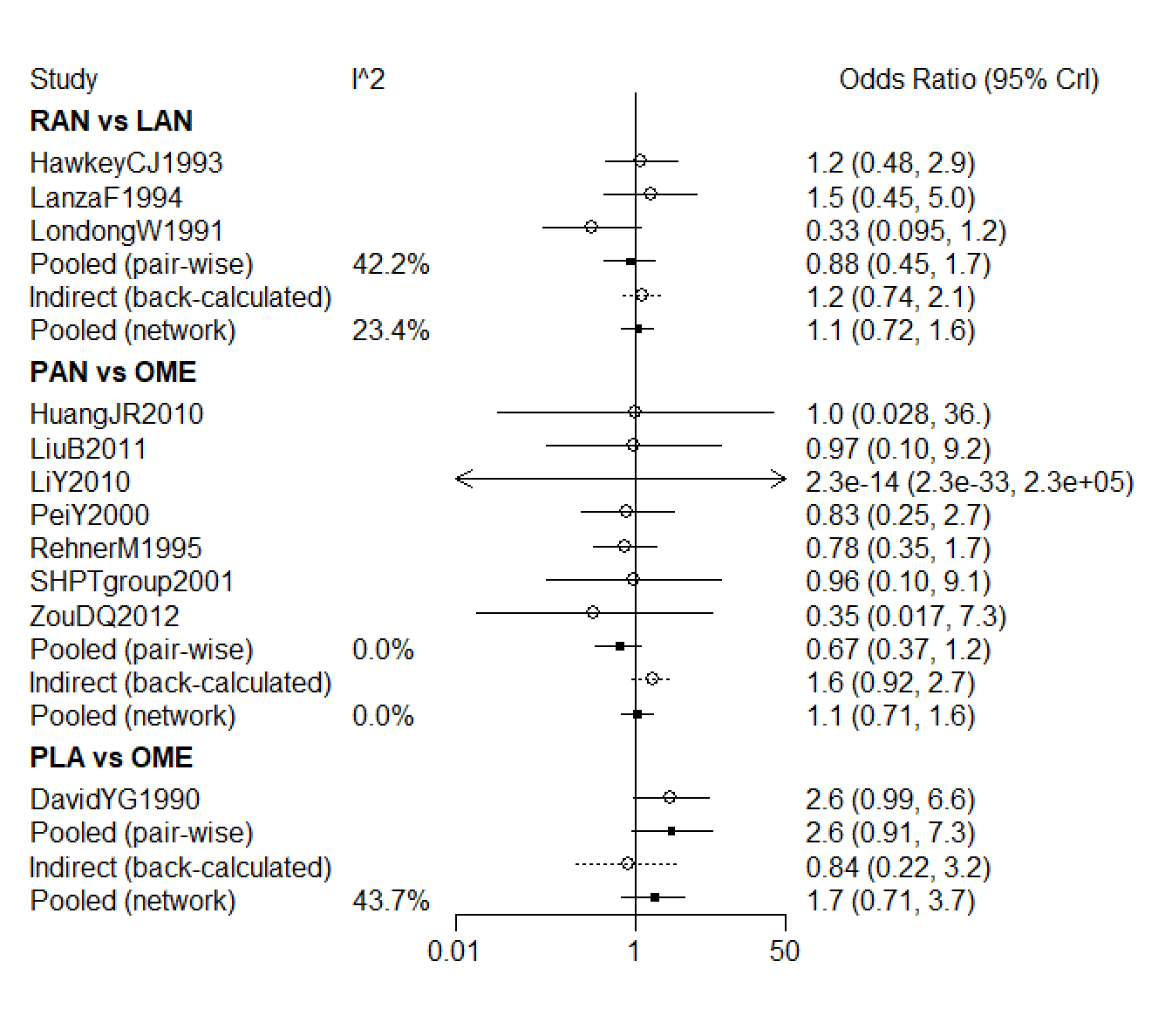


**Figure S4(c). Forest plot of network meta-analysis results for adverse events.**


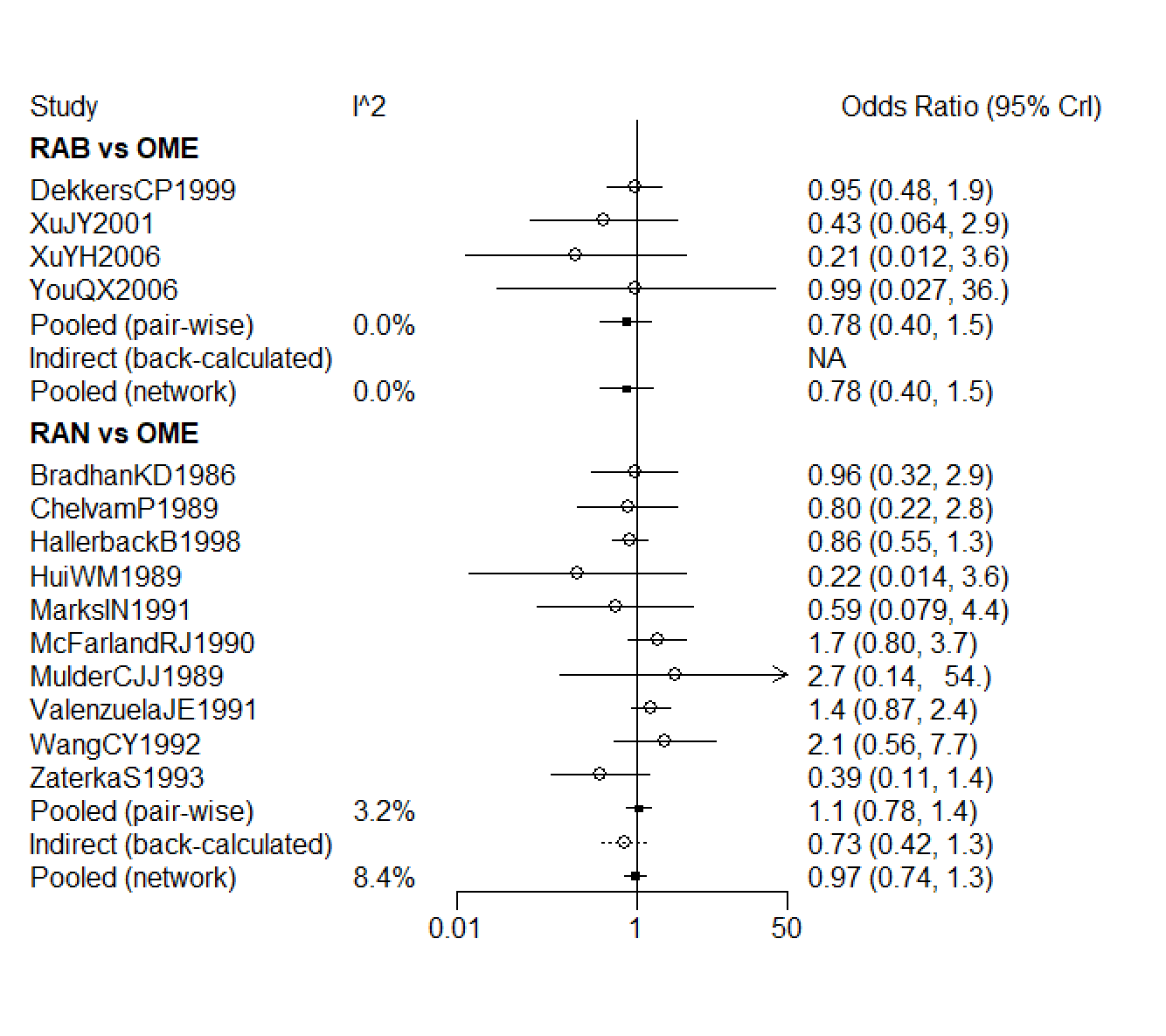


**Figure S4(d). Forest plot of network meta-analysis results for adverse events.**


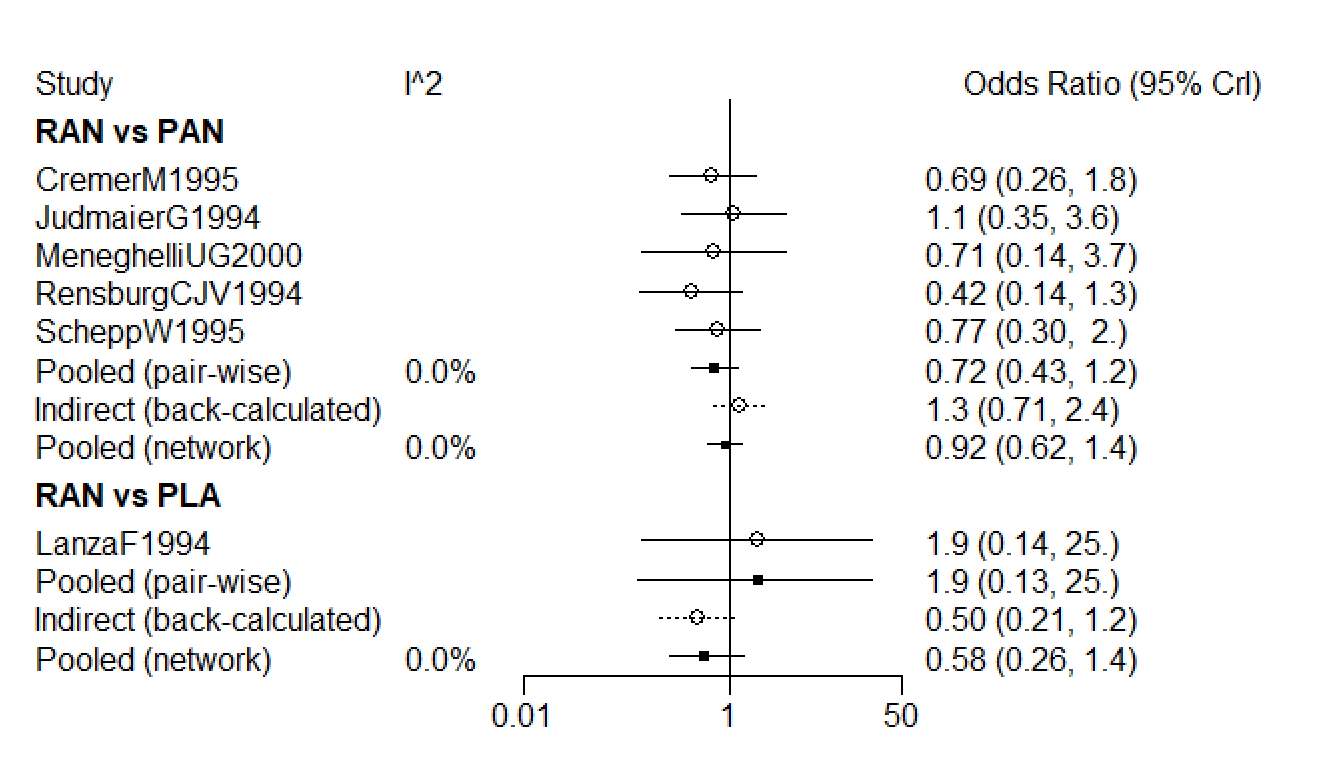


**Figure S4(e). Forest plot of network meta-analysis results for adverse events.**


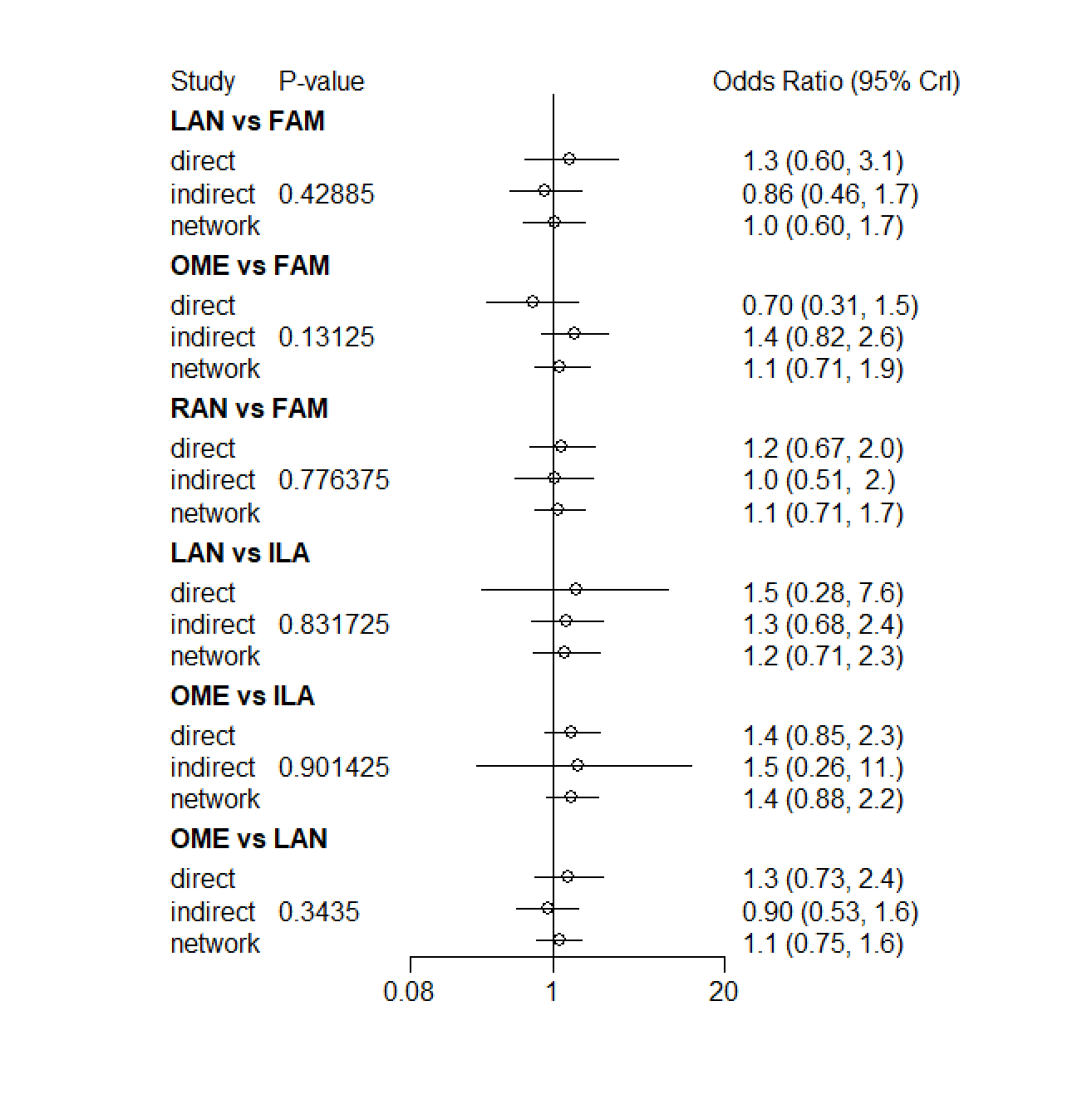


**Figure S5(a). Assessment of network consistency for Adverse events. Only comparisons for which both pairwise and indirect estimates are available.**


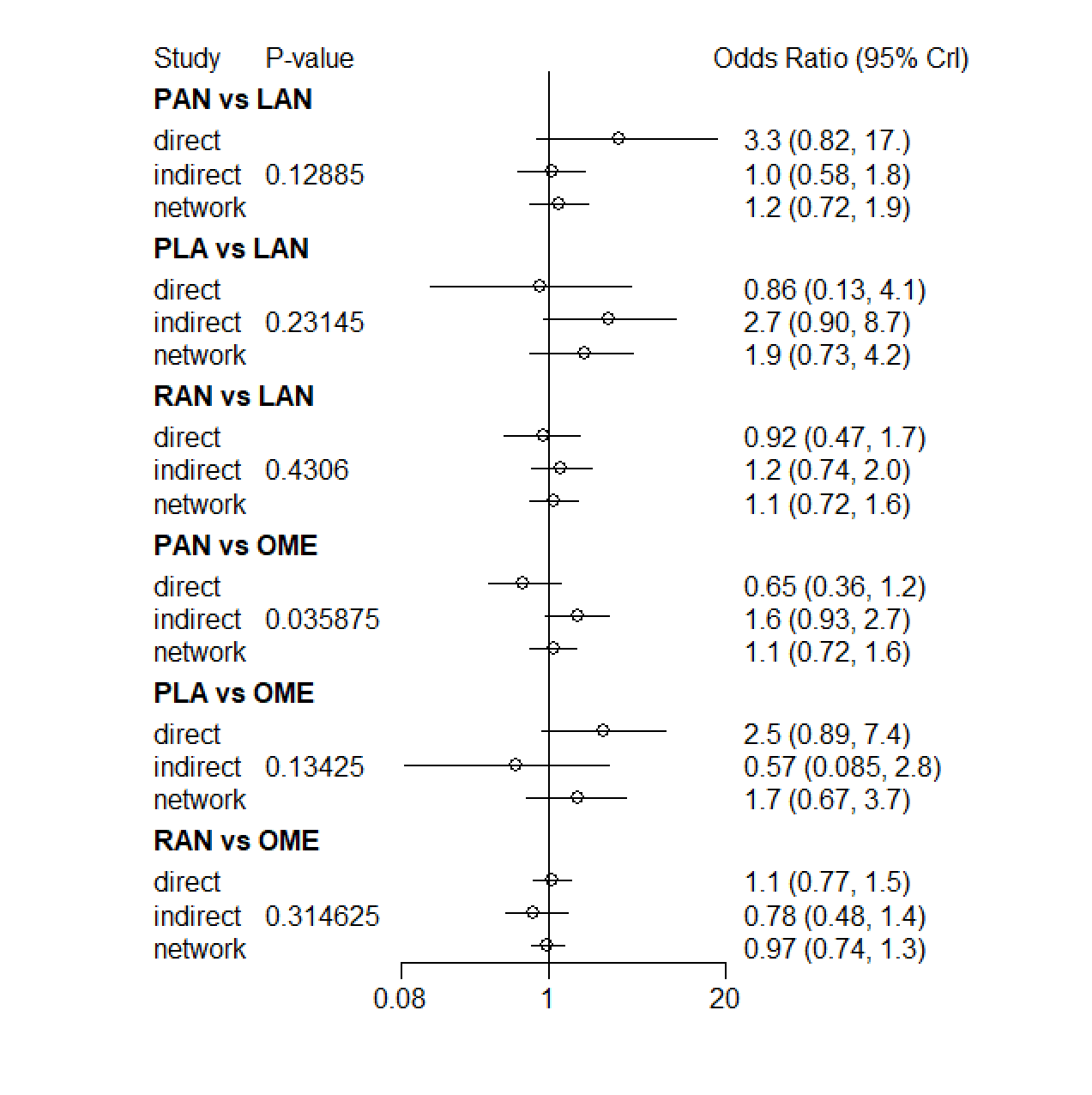


**Figure S5(b). Assessment of network consistency for Adverse events. Only comparisons for which both pairwise and indirect estimates are available.**


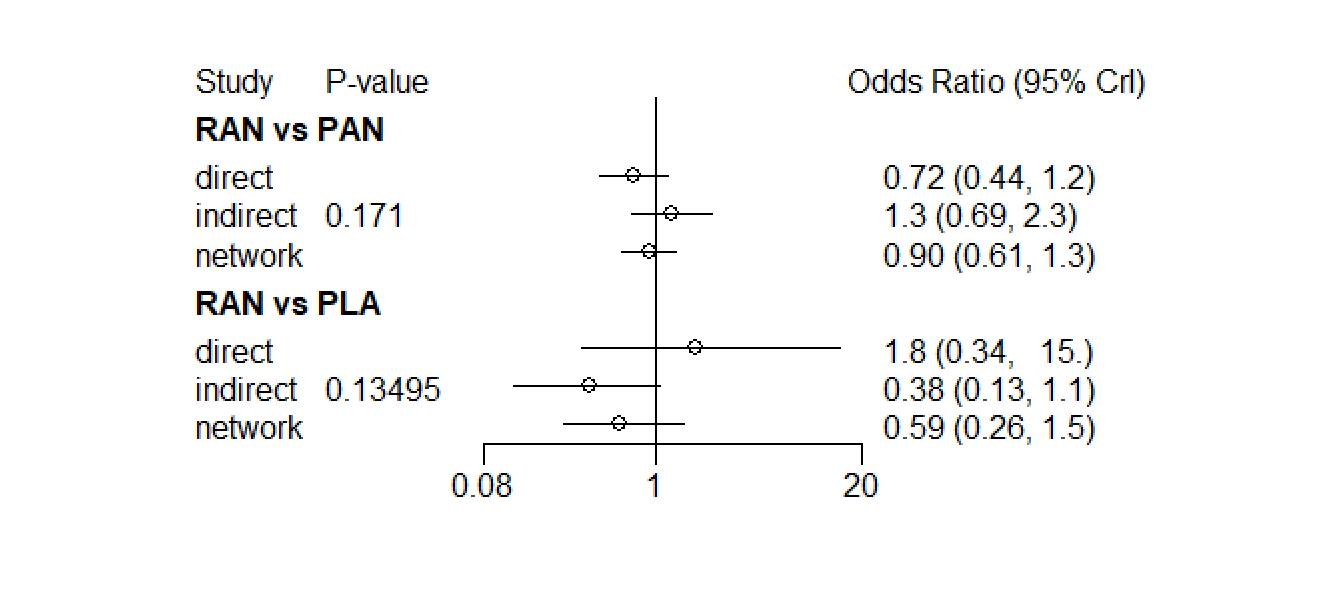


**Figure S5(c). Assessment of network consistency for Adverse events. Only comparisons for which both pairwise and indirect estimates are available.**


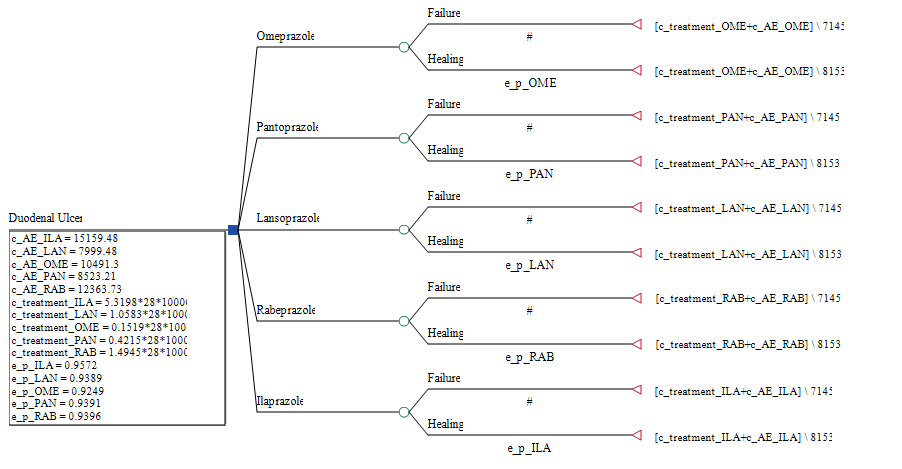


**Figure S6. Decision tree model of standard dose PPIs in short-term treatment of duodenal ulcer over 1 year.**


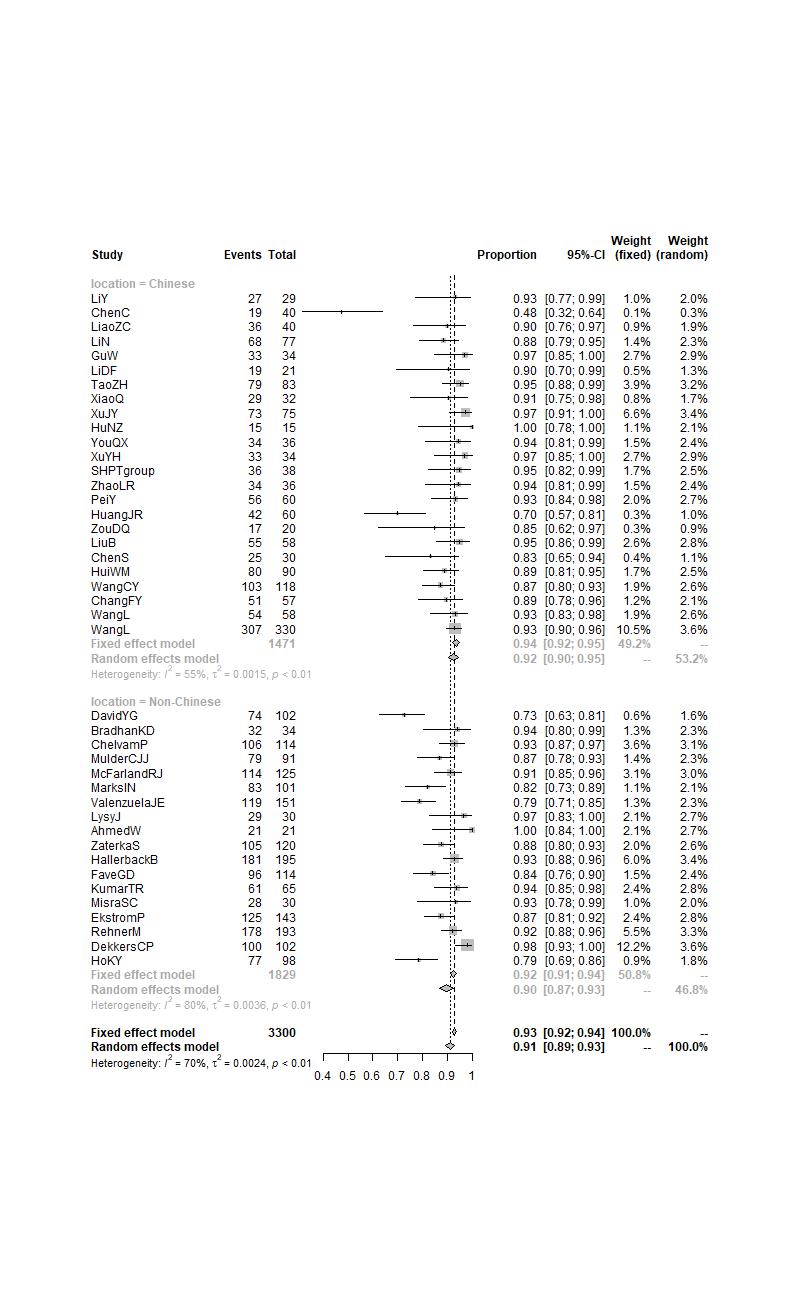


**Figure S7. The 4-week ulcer healing rate of omeprazole based on single-arm meta-analysis.**


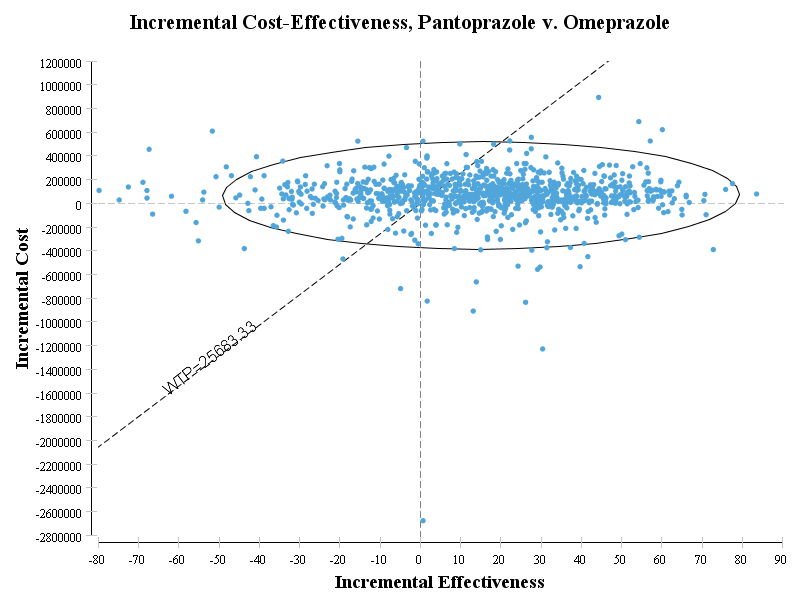


**Figure S8. Probabilistic sensitivity analysis result of pantoprazole vs omeprazole.**


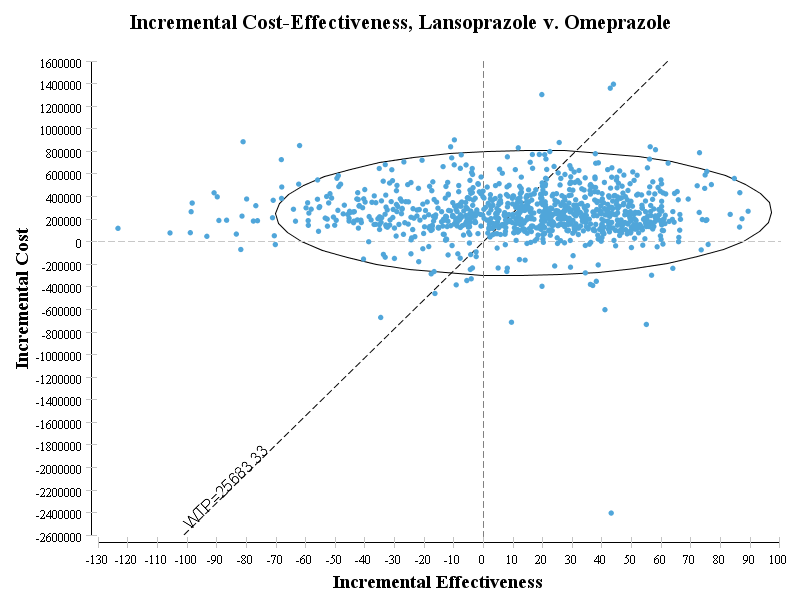


**Figure S9. Probabilistic sensitivity analysis result of lansoprazole vs omeprazole.**


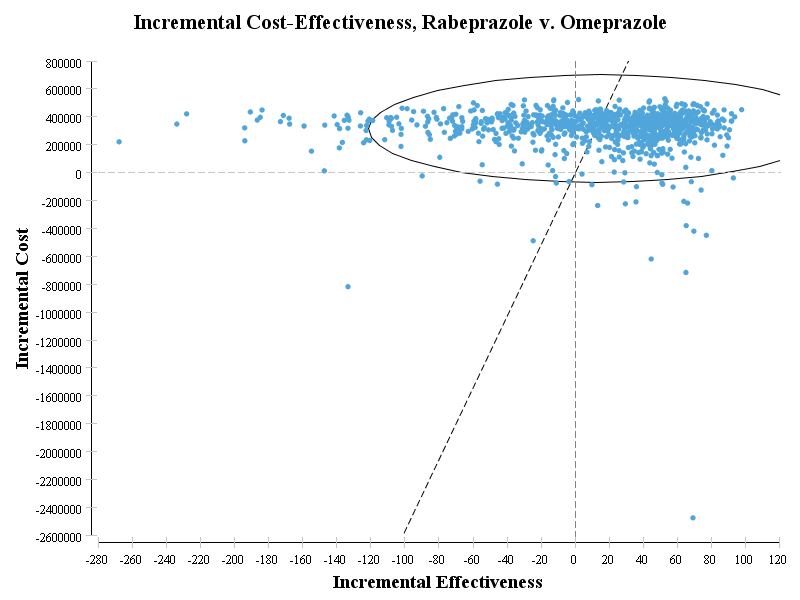


**Figure S10. Probabilistic sensitivity analysis result of rabeprazole vs omeprazole.**


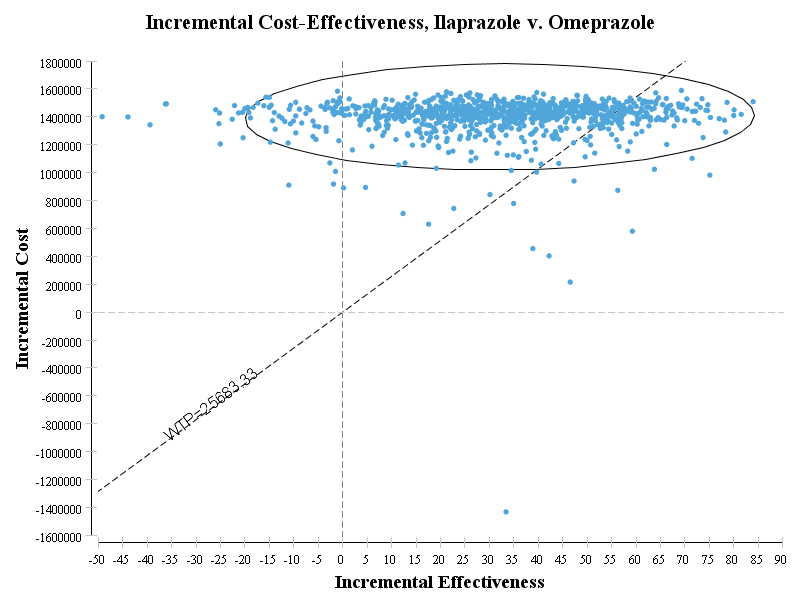


**Figure S11. Probabilistic sensitivity analysis result of ilaprazole vs omeprazole.**
